# Dataset describing two reference models for full-spectral lighting and daylight simulations together with implementations for two software systems

**DOI:** 10.1016/j.dib.2026.112922

**Published:** 2026-06-01

**Authors:** David Geisler-Moroder, Marshal Maskarenj, Greg Ward, Taoning Wang, Eleanor S. Lee, Sergio Altomonte

**Affiliations:** a Unit of Energy Efficient Building, University of Innsbruck, Technikerstrasse 13 6020 Innsbruck, Austria; b UCLouvain, Université catholique de Louvain, Architecture et Climat, Louvain Research Institute for Landscape, Architecture, Built Environment, Place du Levant 1 1348 Louvain-la-Neuve, Belgium; c Anyhere Software, 950 Creston Road, Berkeley, CA 94708, USA; d Energy Technologies Area, Lawrence Berkeley National Laboratory (LBNL), 1 Cyclotron Road, Berkeley, CA 94720, USA

**Keywords:** Simulation models, Spectral simulation, Daylighting, Electric lighting, Non-visual effects, Office, Factory

## Abstract

A dataset of two spectral lighting simulation reference models – one office and one factory hall – is presented. It aims to demonstrate and support full-spectral daylight and electric lighting simulations and facilitate evaluation of non-visual effects of light. The dataset includes Rhino CAD geometry, comprehensive spectral material and light source data and window system BSDF data. Example implementations in the two software tools, Radiance and OWL, enable reproducible workflows and support adoption in other software. The dataset is openly available on Zenodo.

The office model reproduces Room 518 at the University of Innsbruck, including a west-facing façade and interior furnishings. The factory hall model follows the proposed geometry in the European standard 15193 for building energy performance. Interior reflectances in the office were measured in-situ using a handheld spectrometer. Exterior spectra and factory hall materials matching specified reflectances were obtained from an online spectral materials database. Glazing transmittance was derived from IGDB data using LBNL Optics/WINDOW. BSDFs for venetian blinds at various tilt angles, and for a diffusing pane adapted from the Complex Glazing Database, were generated in WINDOW. Luminaires in both models are specified with photometric files (Eulumdat/IES) and lamp spectra (Fluorescent 840, 4000 K LED).

The provided example implementations (Radiance, OWL) include prepared input data and scripts to run first spectral simulations; example results are also included. The dataset is prepared to support reuse by researchers, designers and software developers for method validation, software engineering and comparison, and development of spectral metrics and controls.

Specifications Table

**Table:** 

Subject	Engineering & Materials science
Specific subject area	Lighting and building simulation as part of architectural lighting design.
Type of data	3D geometry: *.3dm, *.obj Text: *.ies, *.ldt, *.csv, *.txt, *.usr, *.epw.url, *.rad, *.mat, *.fmt, *.pts, *.vf, *.dat, *.wea, *.sky, *.aowl, *.sDA_300lx_50p, *.UDI_100–3000, *.ASE_1000lx_250h, *.DGPabove040, *.mEDI, *.hours, *.energy Markup language: *.xml Script files: *.sh Radiance ambient values: *.amb Radiance matrix data: *.vec, *.mtx, *.ill, *.sm xRadiance function files: *.cal Images: *.hsr, *.hdr, *.tif, *.png Radiance octrees: *.oct Grasshopper files: *.gh Radiance mesh: *.rtm
Data collection	Spectral reflectance data were measured with a handheld spectrometer (model: GretagMacbeth Spectrolino) in the office room 518 in the building Technikerstrasse 13 at the University of Innsbruck. Missing spectra (e.g., for glass transmittance), as well as the characteristics of the surfaces in the factory hall, were derived and converted from an online spectral materials database. The scene geometry for both cases was modelled in a 3D CAD software (Rhino 7). The input data for the reference implementation in Radiance were created in a text editor. The OWL reference implementation was set up using the visual programming language Grasshopper in Rhino. The simulation results were created with the two lighting simulation software systems Radiance and OWL, respectively.
Data source location	University of Innsbruck, Technikerstrasse 13, 6020 Innsbruck, Austria
Unit of Energy Efficient Building, Room 518	
47.26° N, 11.34° E	
Data accessibility	The dataset is openly accessible on the EU Open Research Repository Zenodo. Repository name: Zenodo Data identification number: 10.5281/zenodo.18836177 Direct URL to data: https://doi.org/10.5281/zenodo.20344638 Instructions for accessing these data: The data are in zipped folders. Unzip to get the described data files.
Related research article	*None.*

## 1 Value of the Data

Conducting hyperspectral (i.e., N-channel, with *N* > 3) simulations remains challenging due to non-standardized workflows and the in-depth knowledge needed to generate spectrally resolved inputs for the models. Worked examples demonstrate explicitly how these simulations can be done, making the task of learning this skill more accessible, manageable, and easier to comprehend for researchers, lighting practitioners, and professionals with related interests (e.g., health industry).

As with classic three-band RGB simulations, good judgment is required to know how to set the many parameters that define the accuracy and computational speed of hyperspectral simulations. Researchers and practitioners can use these data to learn through testing for reproducibility against their own unique workflow, evaluating trade-offs for their intended context (e.g., speed for early design, accuracy for detailed engineering), and benchmarking results given permutations in parameters of interest. Software developers can also use these data to benchmark new model developments. The provided data includes several intermediate results generated during simulation as well as final results, which all can used to compare own implementations against them.

The datasets span base models for two space types (office and factory hall) and software implementations at two levels of accessibility (command line versus software tool interface) to address the breadth of expertise across the lighting industry. The two common space types can be used for modelling different types of fenestration, lighting, and control strategies.

In detail, the value of the data is:

• The two models contain all the information necessary to perform hyperspectral lighting and daylight simulations. This allows new software systems, or new evaluations, based on spectral data to be tested. This encourages evaluations of non-visual effects of light, which are becoming increasingly relevant in design and standardization.
• With an office model and a factory hall model, two main application areas for lighting and daylighting design are covered (offices account for 23% of the European non-residential building stock, wholesale & retail for 28%). This provides a valuable testbed for software developers and users.
• The example implementations in two software systems, Radiance and OWL, provide a template for the use of the data for later adoption by readers in their own software systems. On the one hand, Radiance, the state-of-the-art software for daylight simulations, which also serves as the basis for many other systems, was selected. On the other hand, OWL, a software that is easily accessible as a Grasshopper application, can be used for further development.
• The implementation in Radiance helps new users and developers to understand Radiance's toolbox-based approach and apply it to its new hyperspectral rendering functionality. The scripts for OWL help users to get started with hyperspectral simulations based on a Grasshopper-based workflow.
• Key stakeholders who can benefit from using these data are software developers, researchers and building designers/engineers, all in the areas of lighting design and research, including non-visual effects of light.

## 2 Background

Daylight and lighting design no longer focus solely on visual requirements (brightness, uniformity, glare protection), but must also take biological or non-visual effects of light into account. These effects encompass physiological and psychological responses to light, regulating circadian rhythm, mood, and alertness through intrinsically photosensitive retinal ganglion cells (ipRGCs) [1]. Because conventional RGB-based simulations are insufficient to evaluate these effects, spectral lighting simulation methods were developed [2,3]. Instead of the conventional three-band RGB simulations used for photopic data, these hyperspectral calculations involve N-wavelength band simulations (e.g., 9–81 bands). With the growing relevance of these non-visual effects of light due to inclusion in standards and building certifications, further implementations in software tools followed and were validated [4].

To support development in this emerging field by providing researchers, users, and developers with templates for hyperspectral simulations, two reference rooms were modelled within the international expert group in IEA SHC Task 70 [5]. These models, including all necessary spectral data, should serve as uniform basis for testing spectral simulation methods and tools, analysing metrics based on spectral data, and testing system and control solutions for spectral daylight and lighting design. Examples of implementations in two software tools that have recently included hyperspectral functionalities are given: Radiance [6] and OWL [7].

## 3 Data Description

The dataset [8] consists of two main components. First, the models of the two reference spaces – an office and a factory hall – consisting of CAD files with 3D geometry and all necessary data for specification in lighting simulation software for spectral simulations. Second, example implementations and result files are provided for the Radiance and OWL software systems.

### 3.1 Data files for the models

#### 3.1.1 Reference room “Office”

The files listed in Table 1 describe the model data for the reference room “Office”. Table 2 describes the spectral transmittance data for the reference room “Office” in folder T70_Office/spectra/. Table 3 describes the spectral reflectance data for the reference room “Office” in folder T70_Office/spectra/. Table 4 describes the spectral power distribution of the fluorescent lamp used in the luminaire in the reference room “Office” in folder T70_Office/spectra/.

**Table 1:** Presentation of the files describing the model data for the reference room “Office”.

Folder	File(s)	Description
T70_Office/	-	Contains 5 subfolders
./geometry/	t70_reference_office_lab518.3dm	3D geometry of the reference room “Office” modelled in Rhino.
	lab518_shading_lines.txt	Shading lines describing the near horizon, far horizon and total (both combined) outdoor obstructions surrounding the building.
./luminaire/	2722–001_98W.ldt	Luminous intensity distribution of the luminaires in the Eulumdat (LDT) file format.
./spectra/	00027_spectral.csv 00027_spectral_sci.csv	Spectral reflectance data for the exterior façade cladding; *_sci.csv only includes the spectrum with specular component included; retrieved from [11].
	00769_spectral.csv 00769_spectral_sci.csv	Spectral reflectance data for the exterior ground; *_sci.csv only includes the spectrum with specular component included; retrieved from [11].
	BauIng_518_Window.usr BauIng_518_Window_t_vis.csv	Spectral data for the window system without blinds defined and calculated in the OPTICS software.
	Fluorescent_840.csv	Spectral power distribution for a typical fluorescent 840 lamp used in the luminaire in the office.
	InterPane_Float_6mm.usr InterPane_Float_6mm_t_vis.csv	Spectral data for the interior glass wall defined and calculated in OPTICS the software.
	T70_office_ceiling.csv	Spectral reflectance data for the acoustic ceiling; measured.
	T70_office_chairs.csv	Spectral reflectance data for the office chairs; measured.
	T70_office_couch.csv	Spectral reflectance data for the couch; measured.
	T70_office_cupboard.csv	Spectral reflectance data for the cupboard; measured.
	T70_office_desk.csv	Spectral reflectance data for the desks; measured.
	T70_office_desk_legs.csv	Spectral reflectance data for the office desk legs; measured.
	T70_office_door.csv	Spectral reflectance data for the door; measured.
	T70_office_floor.csv	Spectral reflectance data for the floor; measured.
	T70_office_frame.csv	Spectral reflectance data for the window frames; measured.
	T70_office_monitors.csv	Spectral reflectance data for the monitors; measured.
	T70_office_walls.csv	Spectral reflectance data for the walls; measured.
./weatherdata/	Download_EnergyPlus_AUT_ Innsbruck.epw.url	Download link for EPW weather data for location Innsbruck.
./window/	BauIng_Fensteraufbau.txt	Output of WINDOW software describing the setup of the window system in the office space for the blind setting +00.
./window/CSV/	Bauing_ILM_Lamelle_XXX.csv	BSDF data for the office window system without blinds (XXX) in CSV format. The file contains 8 data blocks: Solar/Visible, Transmission/Reflection, Front/Back in all combinations. Each data block is in Klems resolution. At the end integral values are included for information, e.g., total transmission visible (Tvis) or solar (Tsol).
	Bauing_ILM_Lamelle_+00.csv	BSDF data in CSV format for the office window system with blinds in horizontal position, i.e., 0 degree slat angle (+00).
	Bauing_ILM_Lamelle_+??.csv	BSDF data in CSV format for the office window system with blinds in outward tilted positions in 5 degree steps, i.e., +?? Stands for +05 to +85 degrees slat angle.
	Bauing_ILM_Lamelle_-??.csv	BSDF data in CSV format for the office window system with blinds in inward tilted positions in 5 degree steps, i.e., -?? Stands for −05 to −85 degrees slat angle.
./window/BSDF/	Bauing_ILM_Lamelle_XXX.xml	BSDF data for the office window system without blinds in XML format to be directly used in simulation software. The file contains the same 8 data blocks as the CSV file.
	Bauing_ILM_Lamelle_+00.xml	BSDF data in XML format for the office window system with blinds in horizontal position.
	Bauing_ILM_Lamelle_+??.xml	BSDF data in XML format for the office window system with blinds in outward tilted positions in 5 degree steps (+05 to +85).
	Bauing_ILM_Lamelle_-??.xml	BSDF data in XML format for the office window system with blinds in inward tilted positions in 5 degree steps (−05 to −85).

**Table 2:** Description and visualization of the spectral transmittance data for the reference room “Office” in folder T70_Office/spectra/.

Geometry	Colour	Transmittance		Spectral data
(layer name in 3D model)		V(λ)	M(λ)	
Glass wall (interior_walls_glass)	Transparent	88.68%	89.32%	
Files: InterPane_Float_6mm.usr / InterPane_Float_6mm_t_vis.csv				
Glazing (glazing)	Transparent	65.23%	66.16%	
Files: BauIng_518_Window.usr / BauIng_518_Window_t_vis.csv

**Table 3:** Description and visualization of the spectral reflectance data for the reference room “Office” in folder T70_Office/spectra/.

Geometry	Colour	Reflectance		Spectral data
(layer name in 3D model)		V(λ)	M(λ)	
Walls (interior_walls)	White	86.88%	85.25%	
File: T70_office_walls.csv				
Ceiling (ceiling)	White	71.84%	64.62%	
File: T70_office_ceiling.csv				
Floor (floor)	Yellow	40.08%	24.32%	
File: T70_office_floor.csv				
Door (door)	White	85.66%	81.73%	
File: T70_office_door.csv				
Desks (desks)	Wood	65.58%	53.85%	
File: T70_office_desk.csv				
Desk legs (desk_legs)	Aluminum	30.78%	30.62%	
File: T70_office_desk_legs.csv				
Chairs (chairs)	Black	2.60%	2.60%	
File: T70_office_chairs.csv				
Cupboard (cupboard)	Light gray	65.55%	59.60%	
File: T70_office_cupboard.csv				
Couch (couch)	Olive green	12.58%	10.38%	
File: T70_office_couch.csv				
Monitors (monitors)	Black	2.35%	2.47%	
File: T70_office_monitors.csv				
Window frame and sill (window_frame)	White, specular finish	86.31%	86.00%	
File: T70_office_frame.csv				
Facade cladding (exterior_facade)	Grey (Grey Aluminium Facade Cladding)	20.01%	18.94%	
File: 00027_spectral_sci.csv				
Exterior ground (exterior_ground)	Grey (Exterior Concrete Floor)	22.02%	21.54%	
File: 00769_spectral_sci.csv

**Table 4:** Description and visualization of the spectral power distribution of the fluorescent lamp used in the luminaire in the reference room “Office” in folder T70_Office/spectra/.

Correlated Colour Temperature	Colour Rendering	Spectral data
CCT	Ra	
4001 K	78	
File: Fluorescent_840.csv		

#### 3.1.2 Reference room “Hall”

The files listed in Table 5 describe the model data for the reference room “Hall”. Table 6 describes the spectral transmittance data for the reference room “Hall” in folder T70_Hall/spectra/. Table 7 describes the spectral reflectance data for the reference room “Hall” in folder T70_Hall/spectra/. Table 8 describes the spectral power distribution of the LED lamp used in the luminaire in the reference room “Hall” in folder T70_Hall/spectra/.

**Table 5:** Presentation of the files describing the model data for the reference room “Hall”.

Folder	File(s)	Description
T70_Hall/	-	Contains 5 subfolders
./geometry/	t70_reference_hall.3dm	3D geometry of the reference room “Hall” modeled in Rhino.
./luminaire/	42188062_product_datasheet _en.pdf	Product data sheet of a CRAFT II performance M LED high-bay luminaire by Zumtobel.
	42188062_(STD_LEO).LDT / *.IES	Luminous intensity distribution of the luminaire in the two file formats Eulumdat (LDT) and IES.
./rooflight/	T70_Reference_Hall_Skylight.txt	Output of WINDOW software describing the setup of the window system in the rooflight.
	T70_Reference_Hall_Skylight.csv	BSDF data for the rooflight window system in CSV format. The file contains 8 data blocks: Solar/Visible, Transmission/Reflection, Front/Back in all combinations. Each data block is in Klems resolution. At the end integral values are included for information, e.g., total transmission visible (Tvis) or solar (Tsol).
	T70_Reference_Hall_Skylight.xml	BSDF data for the rooflight window system in XML format- to be directly used in simulation software. The file contains the same 8 data blocks as the CSV file.
./spectra/	00002_spectral.csv 00002_spectral_sci.csv	Spectral reflectance data for the floor; *_sci.csv only includes the spectrum with specular component included; retrieved from [11].
	00552_spectral.csv 00552_spectral_sci.csv	Spectral reflectance data for the walls; *_sci.csv only includes the spectrum with specular component included; retrieved from [11].
	00677_spectral.csv 00677_spectral_sci.csv	Spectral reflectance data for the rooflight frame; *_sci.csv only includes the spectrum with specular component included; retrieved from [11].
	00995_spectral.csv 00995_spectral_sci.csv	Spectral reflectance data for the ceiling; *_sci.csv only includes the spectrum with specular component included; retrieved from [11].
	CIE_illum_LEDs_03_norm.csv	Spectral power distribution for CIE typical 4000 K LED lamp; retrieved from [17].
	Guardian_ClimaGuard_ doublePane.usr Guardian_ClimaGuard_ doublePane_t_vis.csv	Spectral data for window system defined and calculated in OPTICS software.
	diffusing_shade_generic_60.csv	Generic spectral transmittance for light grey shade with transmittance of 0.60 for all wavelengths; data assumed, generated in text editor.
./weatherdata/	Download_EnergyPlus_DEU_Stuttgart.epw.url	Download link for EPW weather data for location Stuttgart.

**Table 6:** Description and visualization of the spectral transmittance data for the reference room “Hall” in folder T70_Hall/spectra/.

Geometry	Colour	Transmittance		Spectral data
(layer name in 3D model)		V(λ)	M(λ)	
Rooflight glass (factory_rooflight)	Transparent	74.65%	72.54%	
Files: Guardian_ClimaGuard_doublePane.usr / Guardian_ClimaGuard_doublePane_t_vis.csv				
Rooflight diffusing layer (factory_rooflight)	Translucent (diffuse polycarbonate)	60.00%	60.00%	
File: diffusing_shade_generic_60.csv

**Table 7:** Description and visualization of the spectral reflectance data for the reference room “Hall” in folder T70_Hall/spectra/.

Geometry	Colour	Reflectance		Spectral data
(layer name in 3D model)		V(λ)	M(λ)	
Walls (factory_walls)	Neutral, 50%	45.83%	46.30%	
File: 00552_spectral_sci.csv				
Ceiling (factory_ceiling)	Neutral, 70%	72.33%	69.03%	
File: 00995_spectral_sci.csv				
Floor (factory_floor)	Neutral, 20%	20.06%	19.10%	
File: 00002_spectral_sci.csv				
Rooflight frame (factory_rooflight_ frame)	Aluminum	51.54%	51.52%	
File: 00677_spectral_sci.csv

**Table 8:** Description and visualization of the spectral power distribution of the LED lamp used in the luminaire in the reference room “Hall” in folder T70_Hall/spectra/.

Correlated Colour Temperature	Colour Rendering	Spectral data
**CCT**	**Ra**	
4103 K	85	
File: CIE_illum_LEDs_03_norm.csv		

### 3.2 Data files for implementation in Radiance

#### 3.2.1 Reference room “Office”

The data listed in Table 9 contain all necessary files and scripts to run a spectral Radiance simulation of the office model. Additionally, Table 10 lists the files that were created during the simulation.

**Table 9:** Presentation of the files and data structure in the folder *Implementation_Radiance/office/* describing the implementation of the office reference model in Radiance.

File / directory	Description
_office_step00_all.sh	Shell script to run the overall Radiance simulation for the office model. This script subsequently calls all scripts _office_step*.sh.
_office_step*.sh	Separate shell scripts for the single calculation steps 01 to 20.
base_model.rad	Radiance scene description for daylight calculations including the material definition and the scene objects, with luminaires switched off, and without window glazing.
base_model_night.rad	Radiance scene description for electric lighting calculations including the material definition and the scene objects, with luminaires switched on, and with window glazing.
**materials/**	Folder containing file *materials.mat*.
./materials.mat	Spectral Radiance material definition for the objects in the scene.
**misc/**	Folder containing file *glare.fmt*.
./glare.fmt	String format definition of *evalglare* result output.
**objects/**	Folder containing scene objects in Radiance format *.rad.
./ceiling.rad	Geometry definition of office room ceiling.
./ceiling_tiles.rad	Geometry definition of office room ceiling tiles.
./chairs.rad	Geometry definition of chairs in office room.
./couch.rad	Geometry definition of couch in office room.
./cupboard.rad	Geometry definition of cupboard in office room.
./desks.rad	Geometry definition of desks in office room.
./desk_legs.rad	Geometry definition of desk legs in office room.
./door.rad	Geometry definition of office room door.
./facade.rad	Geometry definition of office room facade.
./floor.rad	Geometry definition of office room floor.
./glazing.rad	Geometry definition of office room glazing (window).
./ground.rad	Geometry definition of exterior ground.
./interior_wall.rad	Geometry definition of interior walls of office room.
./interior_wall_bglass.rad	Geometry definition of interior walls of office room.
./interior_wall_glass.rad	Geometry definition of interior glass wall of office room.
./monitors.rad	Geometry definition of interior glass wall of office room, modified with material description to block interior interreflections.
./window_frames.rad	Geometry definition of window frames in office room.
**pts/**	Folder containing sensor points definition files *.pts.
./ceil_sensor.pts	Sensor file describing a look-down sensor mounted centrally on the ceiling.
./facade_sensor.pts	Sensor file describing a vertical sensor mounted at the façade outside the window.
./workplane.pts	Sensor file describing 441 (21 × 21) sensor points on workplane height of 0.75 above the floor.
**sources/**	Folder containing data for the recessed luminaires in the office model and a sky description file.
./2722–001_98W.dat	Radiance luminous intensity distribution data file.
./2722–001_98W.ies	Luminaire IES *.ies photometric data file.
./2722–001_98W.ldt	Luminaire Eulumdat *.ldt photometric data file.
./2722–001_98W.rad	Radiance description of luminaire.
./2722–001_98W_off.rad	Radiance description of luminaire, switched off.
./sky_r4.rad	Sky description in Radiance format using Reinhart -m 4 subdivision.
**spectra/**	Folder containing spectral data for window glazing and lamp, as well as CIE colour matching functions.
./BauIng_518_Window_t_vis.csv	Spectral transmittance data for the window glazing.
./CIE_xyz_1931_2deg.csv	CIE 1931 colour-matching functions, 2 degree observer.
./Fluorescent_840.csv	Spectral power distribution of fluorescent 840 lamp as used in the office luminaire.
./InterPane_Float_6mm_ t_vis.csv	Spectral transmittance data for the interior glass wall.
**weatherdata/**	Folder containing reference weather data.
./ Download_EnergyPlus_AUT_ Innsbruckgart.epw.url	Download link for Energy Plus weather data file for location Innsbruck, Austria.
**window/**	Folder containing BSDF files and Radiance window description.
./window_kf.rad	Geometry definition of office room glazing (window), modified with *glow* material description to act as emitter.
**window/BSDF/**	Subfolder containing BSDF data.
./BSDF/Bauing_ILM_ Lamelle_+75.xml	BSDF data file in Klems resolution for the office window system with blinds in closed position (slat tilt angle 75° to the exterior).
./BSDF/Bauing_ILM_ Lamelle_XXX.xml	BSDF data file in Klems resolution for the office window system without blinds (shading system retracted).

**Table 10:** Presentation of the files and data structure in the folder *Implementation_Radiance/office_with_results/.* Only elements created during the simulation are listed, i.e., additional to Table 9.

File / directory	Description
**amb/**	Folder for ambient files.
./base_model_night.amb	Ambient file for scene base_model_night.rad.
**atmos_data/**	Folder containing atmospheric parameters computed during simulation for the Innsbruck weather tape.
./irrad_ms_mie_ca_*.dat	31 files with parameters for irradiance, summer, aerosol optical depth (*) between 0.05 and 0.38.
./irrad_mw_mie_ca_*.dat	19 files with parameters for irradiance, winter, aerosol optical depth (*) between 0.02 and 0.21.
./scat1m_ms_mie_ca_*.dat	31 files with parameters for Mie scattering, summer, aerosol optical depth (*) between 0.05 and 0.38.
./scat1m_mw_mie_ca_*.dat	19 files with parameters for Mie scattering, winter, aerosol optical depth (*) between 0.02 and 0.21.
./scat_ms_mie_ca_*.dat	31 files with parameters for scattering, summer, aerosol optical depth (*) between 0.05 and 0.38.
./scat_mw_mie_ca_*.dat	19 files with parameters for scattering, winter, aerosol optical depth (*) between 0.02 and 0.21.
./tau_ms_mie_ca_*.dat	31 files with parameters for transmittance, summer, aerosol optical depth (*) between 0.05 and 0.38.
./tau_mw_mie_ca_*.dat	19 files with parameters for transmittance, winter, aerosol optical depth (*) between 0.02 and 0.21.
**mtx/**	Folder with simulation outputs and intermediate calculation results.
./blinds+75.mtx	Spectral BSDF of venetian blinds in closed position (75 tilt angle of slats) including spectral transmittance of glazing.
./ceil_sensor_kf.vec	Relative contributions from window Klems directions to the ceiling sensor.
./daylight_r4.mtx	Daylight matrix D with contributions from sky to window.
./electric_ceil_sensor_ill.txt	Illuminance at ceiling sensor from electric lighting only.
./electric_ill.cal	Helper function with intermediate results.
./electric_light_levels_blinds.txt	Hourly electric lighting levels in case of window with venetian blinds always closed.
./electric_light_levels_control.txt	Hourly electric lighting levels in case of window with venetian blinds controlled.
./electric_light_levels_noblinds.txt	Hourly electric lighting levels in case of window with venetian blinds always open (retracted).
./electric_wp_ill.mtx	Illuminance levels on workplane sensors from electric lighting only.
./electric_wp_ill_avg.txt	Average workplane illuminance from electric lighting only.
./facade_sensor_r4.vec	Daylight contributions per Klems direction at the ceiling sensor.
./Innsbruck_daytime_facade_ext_irrad.txt	Hourly exterior vertical irradiance on façade-mounted sensor.
./Innsbruck_daytime_kf.mtx	Matrix combining daylight matrix and spectral sky matrix.
./Innsbruck_daytime_sky4.mtx	Spectral sky matrix for location Innsbruck, Reinhart -m 4 resolution.
./Innsbruck_daytime_useblinds.txt	Hourly control file when to activate blinds according to threshold 120W/m^2^ of exterior irradiance at façade sensor.
./Innsbruck_day_blinds.mtx	Matrix combining spectral BSDF of blinds, daylight matrix and spectral sky matrix.
./Innsbruck_day_blinds_ wp_ill.mtx	Hourly illuminance levels on workplane sensors from daylight with venetian blinds always closed.
./Innsbruck_day_noblinds.mtx	Matrix combining spectral BSDF of glazing, daylight matrix and spectral sky matrix.
./Innsbruck_day_noblinds_wp_ill.mtx	Hourly illuminance levels on workplane sensors from daylight with venetian blinds always open (retracted).
./Innsbruck_sunonly_blinds.mtx	Matrix combining spectral BSDF of blinds, daylight matrix and spectral sky matrix for sun-only.
./Innsbruck_sunonly_blinds_wp_direct_ill.mtx	Hourly illuminance levels direct-only (without interreflections) and from sun-only on workplane sensors from daylight with venetian blinds always closed.
./Innsbruck_sunonly_kf.mtx	Matrix combining daylight matrix and spectral sky matrix for sun-only.
./Innsbruck_sunonly_noblinds.mtx	Matrix combining spectral BSDF of glazing, daylight matrix and spectral sky matrix for sun-only.
./Innsbruck_sunonly_noblinds_wp_direct_ill.mtx	Hourly illuminance levels direct-only (without interreflections) and from sun-only on workplane sensors from daylight with venetian blinds always open (retracted).
./Innsbruck_sunonly_sky4.mtx	Spectral sky matrix for location Innsbruck, Reinhart -m 4 resolution, direct-sun only.
./noblinds.mtx	Spectral BSDF of window glazing (no venetian blinds).
./wp_direct_kf.mtx	Direct-only view matrix V for illuminance on workplane sensors.
./wp_sensor_kf.mtx	View matrix V for illuminance on workplane sensors.
**mtx/seat?/**	Subfolders for view positions at seat 1, 2 and 3.
./comp*.hsr	Spectral image files for each of the 145 Klems patch contributions from the window to the view (* from 000 to 144).
**oct/**	Folder for octrees.
./base_model_night.oct	Radiance octree for office room scene description with electric lighting only.
**results/**	Folder for results.
./electric_light_blinds.hours	Number of hours with electric light switched on for situation with venetian blinds always closed.
./electric_light_annual_blinds.energy	Annual electricity use [kWh] for electric lighting for situation with venetian blinds always closed.
./electric_light_noblinds.hours	Number of hours with electric light switched on for situation with venetian blinds always open (retracted).
./electric_light_annual_noblinds.energy	Annual electricity use [kWh] for electric lighting for situation with venetian blinds always open (retracted).
./electric_light_control.hours	Number of hours with electric light switched on for situation with venetian blinds controlled.
./electric_light_annual_control.energy	Annual electricity use [kWh] for electric lighting for situation with venetian blinds controlled.
./Innsbruck_day_blinds_wp_ill. sDA_300lx_50p	Spatial daylight autonomy sDA_300lx_50% for situation with venetian blinds always closed.
./Innsbruck_day_blinds_wp_ill. UDI_100–3000	Useful daylight illuminance UDI_100–3000 for situation with venetian blinds always closed.
./Innsbruck_day_noblinds_wp_ill.sDA_300lx_50p	Spatial daylight autonomy sDA_300lx_50% for situation with venetian blinds always open (retracted).
./Innsbruck_day_noblinds_wp_ill.UDI_100–3000	Useful daylight illuminance UDI_100–3000 for situation with venetian blinds always open (retracted).
./Innsbruck_sunonly_blinds_wp_direct_ill.ASE_1000lx_250h	Direct-only contribution from sun as proxy for Annual Sunlight Exposure ASE_1000lx_250h (but calculated with BSDF, i.e., including scattering in system) for situation with venetian blinds always closed.
./Innsbruck_sunonly_noblinds_wp_direct_ill.ASE_1000lx_250h	Direct-only contribution from sun as proxy for Annual Sunlight Exposure ASE_1000lx_250h (but calculated with BSDF, i.e., including scattering in system) for situation with venetian blinds always open (retracted).
./night_seat2.mEDI	Melanopic equivalent daylight illuminance mEDI at seat 2 for situation with electric lighting only.
./night_seat2.hdr	HDR Radiance image of scene with electric lighting only.
./night_seat2.hsr	Hyperspectral Radiance image of scene with electric lighting only.
./night_seat2.tif	Tiff image of scene with electric lighting only.
**results/anim/**	Subfolder with annual image results and glare evaluations.
**results/anim/seat?/**	Subfolder for view positions at seat 1, 2 and 3.
./blinds_evg.DGPabove040	Percentage of hours with daylight glare probability DGP above threshold of 0.40 for situation with venetian blinds always closed.
./blinds_evg.txt	Hourly *evalglare* results for situation with blinds closed.
./noblinds_evg.DGPabove040	Percentage of hours with daylight glare probability DGP above threshold of 0.40 for situation with venetian blinds always open (retracted).
./noblinds_evg.txt	Hourly *evalglare* results for situation with blinds open (retracted).
**results/anim/seat?/blinds/**	Subfolder for result images of situation with blinds closed.
./frame????.hdr	Hourly result images for 4015 daytime hours (08–18 daily; * from 0000 to 4014).
**results/anim/seat?/noblinds/**	Subfolder for result images of situation with blinds open (retracted).
./frame????.hdr	Hourly result images for 4015 daytime hours (08–18 daily; * from 0000 to 4014).
**spectra/**	Folder with spectral data and helper functions.
./BauIng_518.cal	Spectral transmittance data for the window glazing as callable function.
./BauIng_518_factor.cal	Helper function with intermediate results.
./BauIng_518_smult_780–380.txt	Spectral transmittance data for the window glazing in 20 equally spaced wavelength bands from 780 nm to 380 nm.
./cie31_2deg.cal	CIE 1931 colour-matching functions, 2 degree observer, as callable function.
./Fluorescent_840.cal	Spectral power distribution of fluorescent 840 lamp as used in the office luminaire as callable function.
./Fluorescent_840_factor.cal	Helper function with intermediate results.
./Fluorescent_840_ smult_380–780_81.txt	Spectral power distribution of fluorescent 840 lamp from 380 nm to 780 nm in 5 nm steps.
**weatherdata/**	Folder containing reference weather data.
./AUT_Innsbruck.111200_ IWECday.epw.RemovedDueToLicense	Energy Plus weather data file for location Innsbruck, Austria, including only hours 08 – 18. (The generated data file was removed from the output due to License issues.)

#### 3.2.2 Reference room “Hall”

The data listed in Table 11 contain all the necessary files and scripts to run a spectral Radiance simulation of the factory hall model. Additionally, Table 12 lists the files that were created during the simulation.

**Table 11:** Presentation of the files and data structure in the folder *Implementation_Radiance/hall/* describing the implementation of the factory hall reference model in Radiance.

File / directory	Description
_hall_step00_all.sh	Shell script to run the overall Radiance simulation for the hall model. This script subsequently calls all scripts _hall_step*.sh.
_hall_step*.sh	Separate shell scripts for the single calculation steps 01 to 17.
base_model.rad	Radiance scene description for daylight calculations including the material definition and the scene objects, without luminaires and without rooflight glazing.
base_model_night.rad	Radiance scene description for electric lighting calculations including the material definition and the scene objects, with luminaires switched on, and with rooflight glazing.
**materials/**	Folder containing file *materials.mat*.
./materials.mat	Spectral Radiance material definition for the objects in the scene.
**misc/**	Folder containing file *glare.fmt*.
./glare.fmt	String format definition of *evalglare* result output.
**objects/**	Folder containing scene objects in Radiance format *.rad.
./factory_ceiling.rad	Geometry definition of factory hall ceiling.
./factory_floor.rad	Geometry definition of factory hall floor.
./factory_roof_light.rad	Geometry definition of factory hall rooflight glazing.
./factory_roof_light_ frames.rad	Geometry definition of factory hall rooflight glazing frames.
./factory_walls.rad	Geometry definition of factory hall walls.
./rooms_ceiling.rad	Geometry definition of ceiling of adjacent administrative rooms.
./rooms_floor.rad	Geometry definition of floor of adjacent administrative rooms.
./rooms_walls.rad	Geometry definition of walls of adjacent administrative rooms.
**pts/**	Folder containing sensor points definition files *.pts.
./ceil_sensor.pts	Sensor file describing a look-down sensor mounted centrally on the ceiling.
./workplane.pts	Sensor file describing 728 (28 × 26) sensor points on workplane height of 0.75 above the floor.
**rooflight/**	Folder containing BSDF data.
./T70_Reference_Hall_Skylight.xml	BSDF data file in Klems resolution for the factory hall rooflight glazing with scattering layer.
**sources/**	Folder containing data for the recessed luminaires in the office model and a sky description file.
./42188062_(STD_LEO).dat	Radiance luminous intensity distribution data file.
./42188062_(STD_LEO).ies	Luminaire IES *.ies photometric data file.
./42188062_(STD_LEO).rad	Radiance description of luminaire.
./factory_luminaires.rad	Geometry definition of 64 luminaires in the hall.
./sky_r4.rad	Sky description in Radiance format using Reinhart -m 4 subdivision.
**spectra/**	Folder containing spectral data for rooflight glazing and lamp, as well as CIE colour matching functions.
./CIE_illum_LEDs_03_ norm.csv	Spectral power distribution of CIE typical LED 03, normalized to a maximum value of 1.0.
./CIE_xyz_1931_2deg.csv	CIE 1931 colour-matching functions, 2 degree observer.
./Guardian_ClimaGuard_ doublePane_t_vis.csv	Spectral transmittance data for the rooflight glazing.
**views/**	Folder containing view specifications.
./heast.vf	Radiance view definition for a hemispherical fisheye view, from the centre of the hall at height 1.7 m, view direction East.
./hnorth.vf	Radiance view definition for a hemispherical fisheye view, from the centre of the hall at height 1.7 m, view direction North.
./hsouth.vf	Radiance view definition for a hemispherical fisheye view, from the centre of the hall at height 1.7 m, view direction South.
./hwest.vf	Radiance view definition for a hemispherical fisheye view, from the centre of the hall at height 1.7 m, view direction West.
**weatherdata/**	Folder containing reference weather data.
./ Download_EnergyPlus_DEU_Stuttgart.epw.url	Download link for Energy Plus weather data file for location Stuttgart, Germany.
**window/**	Folder containing BSDF files and Radiance window description.
./roof_skylight.rad	Geometry definition of factory hall rooflight glazing, modified with *glow* material description to act as emitter.

**Table 12:** Presentation of the files and data structure in the folder *Implementation_Radiance/hall_with_results/*. Only elements created during the simulation are listed, i.e., additional to Table 11.

File / directory	Description
**amb/**	Folder for ambient files.
./base_model_night.amb	Ambient file for scene base_model_night.rad
**atmos_data/**	Folder containing atmospheric parameters computed during simulation for the Innsbruck weather tape.
./irrad_ms_mie_ca_*.dat	14 files with parameters for irradiance, summer, aerosol optical depth (*) between 0.14 and 0.27.
./irrad_mw_mie_ca_*.dat	15 files with parameters for irradiance, winter, aerosol optical depth (*) between 0.05 and 0.19.
./scat1m_ms_mie_ca_*.dat	14 files with parameters for Mie scattering, summer, aerosol optical depth (*) between 0.14 and 0.27.
./scat1m_mw_mie_ca_*.dat	15 files with parameters for Mie scattering, winter, aerosol optical depth (*) between 0.05 and 0.19.
./scat_ms_mie_ca_*.dat	14 files with parameters for scattering, summer, aerosol optical depth (*) between 0.14 and 0.27.
./scat_mw_mie_ca_*.dat	15 files with parameters for scattering, winter, aerosol optical depth (*) between 0.05 and 0.19.
./tau_ms_mie_ca_*.dat	14 files with parameters for transmittance, summer, aerosol optical depth (*) between 0.14 and 0.27.
./tau_mw_mie_ca_*.dat	15 files with parameters for transmittance, winter, aerosol optical depth (*) between 0.05 and 0.19.
**mtx/**	Folder with simulation outputs and intermediate calculation results.
./ceil_sensor_kf.vec	Relative contributions from rooflight Klems directions to the ceiling sensor.
./daylight_r4.mtx	Daylight matrix D with contributions from sky to rooflight.
./electric_ceil_sensor_ill.txt	Illuminance at ceiling sensor from electric lighting only.
./electric_ill.cal	Helper function with intermediate results.
./electric_light_levels.txt	Hourly electric lighting levels for daylight-dependent dimmed lighting.
./electric_wp_ill.mtx	Illuminance levels on workplane sensors from electric lighting only.
./electric_wp_ill_avg.txt	Average workplane illuminance from electric lighting only.
./SpecSkylight.mtx	Spectral BSDF of rooflight glazing system.
./Stuttgart_day_skylight.mtx	Matrix combining spectral BSDF of rooflight system, daylight matrix and spectral sky matrix.
./Stuttgart_day_wp_ill.mtx	Hourly daylight illuminance levels on workplane sensors.
./Stuttgart_daytime_kf.mtx	Matrix combining daylight matrix and spectral sky matrix.
./Stuttgart_daytime_sky4.mtx	Spectral sky matrix for location Stuttgart, Reinhart -m 4 resolution.
./Stuttgart_sunonly_kf.mtx	Matrix combining daylight matrix and spectral sky matrix for sun-only.
./Stuttgart_sunonly_sky4.mtx	Spectral sky matrix for location Innsbruck, Reinhart -m 4 resolution, direct-sun only.
./Stuttgart_sunonly_skylight.mtx	Matrix combining spectral BSDF of rooflight system, daylight matrix and spectral sky matrix for sun-only.
./Stuttgart_sunonly_wp_direct_ill.mtx	Hourly sunlight illuminance levels direct-only (without interreflections) and sun-only (no sky) on workplane sensors.
./wp_direct_kf.mtx	Direct-only view matrix V for illuminance on workplane sensors.
./wp_sensor_kf.mtx	View matrix V for illuminance on workplane sensors.
mtx/h*/	Subfolders for view directions * in east, north, south, west.
./comp*.hsr	Spectral image files for each of the 145 Klems patch contributions from the rooflight to the view (* from 000 to 144).
**oct/**	Folder for octrees.
./base_model_night.oct	Radiance octree for factory hall scene description with electric lighting only.
**results/**	Folder for results.
./electric_light.hours	Number of hours with electric light switched on.
./electric_light_annual.energy	Annual electricity use [kWh] for electric lighting.
./night_hnorth.hdr	HDR Radiance image of scene with electric lighting only, view direction North.
./night_hnorth.hsr	Hyperspectral Radiance image of scene with electric lighting only, view direction North.
./night_hnorth.tif	Tiff image of scene with electric lighting only, view direction North.
./night_hnorth.mEDI	Melanopic equivalent daylight illuminance mEDI for view direction North for situation with electric lighting only.
./Stuttgart_day_wp_ill. sDA_300x_50p	Spatial daylight autonomy sDA_300lx_50%.
./Stuttgart_day_wp_ill. UDI_100–3000	Useful daylight illuminance UDI_100–3000.
./Stuttgart_sunonly_wp_direct_ill. ASE_1000lx_250h	Direct-only contribution from sun as proxy for Annual Sunlight Exposure ASE_1000lx_250h (but calculated with BSDF, i.e., including scattering in system).
**results/anim/**	Subfolder with annual image results and glare evaluations.
./h*_evg.DGPabove040	Percentage of hours with daylight glare probability DGP above threshold of 0.40 for view * (east, north, south, west).
./h*_evg.txt	Hourly *evalglare* results for view * (east, north, south, west).
results/anim/h*/	Subfolder for view directions * in east, north, south, west.
./frame????.hdr	Hourly result images for 4015 daytime hours (08–18 daily; * from 0000 to 4014).
**spectra/**	Folder with spectral data and helper functions.
./cie31_2deg.cal	CIE 1931 colour-matching functions, 2 degree observer, as callable function.
./CIE_illum_LEDs_03_norm.cal	Spectral power distribution of CIE typical LED 03 as used in the office luminaire as callable function.
./CIE_illum_LEDs_03_norm _ factor.cal	Helper function with intermediate results.
./CIE_illum_LEDs_03_norm_ smult_380–780_81.txt	Spectral power distribution of CIE typical LED 03 from 380 nm to 780 nm in 5 nm steps.
./Guardian.cal	Spectral transmittance data for the rooflight glazing as callable function.
./Guardian_factor.cal	Helper function with intermediate results.
./Guardian_smult_780–380.txt	Spectral transmittance data for the rooflight glazing in 20 equally spaced wavelength bands from 780 nm to 380 nm.
**weatherdata/**	Folder containing reference weather data.
./DEU_Stuttgart.107380_ IWECday.epw.RemovedDueToLicense	Energy Plus weather data file for location Stuttgart, Germany, including only hours 08 – 18. (The generated data file was removed from the output due to License issues.)

### 3.3 Data files for implementation in OWL

The files and data structure presented in Table 13 describe the implementation of the reference models in OWL. The following tables, Table 14, Table 15, Table 16, Table 17, Table 18, Table 19, Table 20, Table 21, present the files describing the implementation of the reference models in OWL. The following placeholders are used in the names:

• {main}: Main folder for the evaluations, always in "C:\OWL\annuowl".
• {case}: user defined case-name, e.g.: "DiB_Hall".
• {layer}: name of each geometric layer having a specific material-type, e.g.: "SHC70C_H_R_Walls".
• {*}: All view directions: H, N, E, S, W.
• {@}: Files that are re-generated within occupant-centered and grid-based pipelines.
• {^}: Vertical orientations in cardinal directions: N, E, S, W.
• {£}: cluster index for electric luminaires, e.g. 1,2, 3.
• {$}: mid-value CCT index for each user-defined bin.
• {#}: 3-channel segment within the 81-channel spectral range.

**Table 13:** Presentation of the files and data structure describing the implementation of the reference models in OWL.

File / directory	Description
DiB_OWL_Hall.gh	Rhino Grasshopper file to replicate OWL-based simulations for “Hall” case.
DiB_OWL_Hall_Cached.gh	Rhino Grasshopper file to display outcomes of pre-simulated “Hall” case, using data from *C:\OWL\annuowl\DiB_Hall* folder.
DiB_OWL_Office.gh	Rhino Grasshopper file to replicate OWL-based simulations for “Office” case.
DiB_OWL_Office_Cached.gh	Rhino Grasshopper file to display outcomes of pre-simulated “Office” case, using data from *C:\OWL\annuowl\DiB_Ofc* folder.
DiB_Hall/	Folder created from OWL, containing files and subfolders created during the simulation for the Hall reference room. This folder must be placed under *C:\OWL\annuowl* directory, to be readily visualized using *_Cached.gh files.
DiB_Ofc/	Folder created from OWL, containing files and subfolders created during the simulation for the Office reference room.
Supplementary _Data/	Folder to be placed in *C:\OWL\annuowl* directory. Contains additional files necessary for running live simulation (e.g., weather file, spectral data, luminaire data).

**Table 14:** Files for occupant-centered evaluations within the {main}\{case}\ folder.

File	Description
materials.rad	Definitions for reflectance/transmittance in Radiance 3-channel format, combined for all materials in the scene
geom_pointsHd.csv	Position for each user-defined observer in the scene, in cartesian coordinates.
{case}_{layer}.obj	Material-separated geometry exported from grasshopper
ViewPts{*}.txt	Observer positions and view vectors
spectralSkyglow.sky ^@^	Generated sky definition in the Radiance format
{case}_{layer}.rad ^@^	Layer-specific interim Radiance geometry file
{case}_{layer}.rtm ^@^	Layer-specific interim file in Radiance Triangular Mesh format
materialBlack.rad ^@^	Definition of a black material (no reflectance) for supporting Radiance DDS calculations
roomBlack.rad ^@^	Geometry combined with black material
scene.rad ^@^	File combining all RTM-geometry
scene.oct ^@^	Radiance octree for total sky scene
sceneBlack.oct ^@^	Radiance octree for direct-only sky contributions (to be subtracted, to avoid double counting in 2-phase DDS evaluations)
suns.rad ^@^	Definition of sun position and intensity value
sunCoefficientsDDS.oct ^@^	Radiance octree for direct sun evaluations, for sharp and accurate shadows
{case}_illum{*}.mtx	Daylight coefficient matrix for total sky
{case}_illum{*}_B.mtx	Daylight coefficient matrix for direct-only sky
cdsDDS_{*}.mtx	Daylight coefficient matrix for direct sun
location.wea ^@^	Weather file translated from the EPW file for any given global location, in DAYSIM-style format
location.smx ^@^	Sky matrix for total sky
location_B.smx ^@^	Sky matrix for direct-only sky
location_sunM6.smx ^@^	Sky matrix for sun-locations
annualR_{*}.mtx	Annual 3-channel spectral contributions for total sky for each orientation
annualR_{*}B.mtx	Annual 3-channel spectral contributions for direct-only sky for each orientation
annualR_{*}B.ill	Annual hourly sky illuminance for direct-only sky for each orientation
annualR_sun_{*}.ill	Annual hourly direct-solar illuminance for each orientation
annualR_tot{*}.ill	Annual hourly illuminance using DDS logic for each orientation
annualillum{*}.csv	Annual hourly illuminance for each orientation, in a CSV format
ann_CS_{^}.csv	Annual hourly circadian stimulus (CS) values for each vertical orientation
ann_DGP_{^}.csv	Simplified DGP (sDGP) for all annual hours for each vertical orientation
CSA_{^}.csv	Annual hourly 365 × 24 matrix for CS for a user-defined observer location in each vertical location
CS_{^}_graph.png	CS plotted for each vertical orientation, for a user-defined observer location, across 365 × 24 annual daily hours
DGPA_{^}.csv	Annual hourly 365 × 24 matrix of sDGP values for a user-defined observer location in each vertical location
DGP_{^}_graph.png	sDGP plotted for each vertical orientation, for a user-defined observer location, across 365 × 24 annual daily hours
Ch_graph.png	Annual outdoor horizontal hourly CCT values (in Kelvins), calculated via spectral sky models from hemispherical (Perez Sky) luminance.
Cz_graph.png	Annual outdoor horizontal hourly CCT values (in Kelvins), based on Zenith luminance.

**Table 15:** Files for grid-based evaluations within the {main}\{case}\ folder.

File	Description
floorgrid.3dm	3D model representing floor surfaces identified for grid-based evaluation
GrdPtsH.txt	Coordinates for sensor points on the grid.
{case}_ill_Hrz.mtx	Daylight coefficient matrix for total sky, across the grid-sensors
{case}_ill_Hrz_B.mtx	Daylight coefficient matrix for direct-only sky, across the grid-sensors
cdsDDS_Hrz.mtx	Daylight coefficient matrix for direct sun, across the grid-sensors
annualR_Hrz.mtx	Annual 3-channel spectral contributions for total sky, across the grid-sensors
annualR_HrzB.mtx	Annual 3-channel spectral contributions for direct-only sky, across the grid-sensors
annualR_Hrz.ill	Annual hourly total-sky illuminance across the sensor grid
annualR_HrzB.ill	Annual hourly sky illuminance for direct-only sky across the grid
annualR_sun_Hrz.ill	Annual hourly direct-solar illuminance across the grid
annualR_totHrz.ill	Annual hourly illuminance across the sensor grid, following DDS logic
annualillum_Hrz.csv	Annual hourly illuminance across the sensor grid, in CSV format
ann_metrics.csv	Synthesis of Climate Based Daylight Metrics: Daylight Autonomy, continuous Daylight Autonomy, Useful Daylight Illuminance, Annual Sun Exposure, etc. (across annual hours) for each grid point

**Table 16:** Files for evaluation of daylight factors within the {main}\{case}\ folder.

File	Description
skyCIEOvercast.rad	Radiance-format source file with uniform glow to approximate a simplified overcast sky
sceneOC.oct	Radiance octree combining source and full scene geometry (from scene.rad)
indoorDF.txt	Indoor illuminance at each sensor point on the grid from the uniform source
outdoor_pt.txt	Illuminance at an outdoor reference point from the uniform source
daylight_factor.csv	Percentage ratio of indoor illuminance across each grid point, with the outdoor unobstructed horizontal illuminance. Saved for each sensor point in a CSV format

**Table 17:** Files for grid-based evaluations of electric lighting within the {main}\{case}\ folder.

File	Description
all_lum{£}.rad	Radiance definition of electric luminaire within a specific cluster
scenelum{£}.oct	Radiance octree combining material data, scene data, and luminaire definitions
resultsEL_cluster{£}.ill	3-channel irradiance from the luminaire cluster across all grid-based sensor, at peak emission intensity.
resultsEL_cluster{£}_LUX.csv	Horizontal illuminance contribution for each grid-point due to the cluster at peak emission intensity
resultsEL_cluster{£}_RGB.csv	Horizontal 3-channel irradiance contribution for each grid-point due to the cluster at peak emission intensity
DimmingPlot.png	Plot demonstrating optimized schedules for dimming controls against each luminaire cluster, based on desired illuminance at any given sensor position, for annual hours

**Table 18:** Files for multispectral simulations within the {main}\{case}\Spct subfolder.

File	Description
{case}_{layer}.obj	Same as above
ViewPts{*}.txt	Same as above
suns.rad	Same as above
location.wea	Same as above
CCT_bin.csv, CCT_bin_av.csv	Key indexing files for spectral sub-evaluation and data merging
location_head.wea	header lines from the WEA file
location_mod.wea	Body content for annual hours from WEA file
location_{$}.wea	A version of WEA file containing inputs for annual hours when sky spectra was within the {$} CCT bin, demarcated by average value of the bin, e.g. 4500K

**Table 19:** Files for simultaneous evaluation within 27 nested subfolders {main}\{case}\Spct\div{#}. Each subfolder represents spectral irradiance evaluated for one 3-channel segment of the 81-channel spectra.

File	Description
materials.rad	3-channel segment of the 81-channel material reflectance/transmittance data, for all layered materials, corresponding to the subfolder
spectralSkyglow.sky	Same as earlier
{case}_{layer}.obj	Same as earlier, replicated in each nested subfolder
{case}_{layer}.rad	Same as earlier
{case}_{layer}.rtm	Same as earlier but combining respective segment of 81-channnel material definition with RAD geometries
materialBlack.rad	Same as earlier
roomBlack.rad	Same as earlier
scene.rad	Same as earlier, yet with segment of 81-channel material definitions
suns.rad	Same as earlier
ViewPts{*}.txt	Same as earlier
scene.oct	Same as earlier, yet with segment of 81-channel material definitions
sceneBlack.oct	Same as earlier
sunCoefficientsDDS.oct	Same as earlier
{case}_illum{*}.mtx	Same as earlier, yet with segment of 81-channel material definitions
{case}_illum{*}_B.mtx	Same as earlier
cdsDDS_{*}.mtx	Same as earlier
location_{$}.wea	Subset of the WEA file: with $ corresponding to the mid-CCT value within each user-defined CCT bin
location.smx	Interim total-sky matrix file for specific hours (corresponding to the $ bin)
location_B.smx	Interim direct-sky matrix file for specific hours (corresponding to the $ bin)
annualR_{*}.mtx	Interim 3-channel spectral contributions for total sky across orientation (*), for annual hours corresponding to $ bin
annualR_{*}B.mtx	Interim 3-channel spectral contributions for direct-only sky across orientation (*), for annual hours corresponding to $ bin
location_sunM6.smx	Same as earlier, yet for annual hours corresponding to $ bin
annualR_{*}.ill	Sky illuminance (interim file) from total sky for orientation (*), for annual hours corresponding to $ bin
annualR_{*}B.ill	Sky illuminance (interim file) from direct-only sky for orientation (*), for annual hours corresponding to $ bin
annualR_sun_{*}.ill	Direct solar illuminance (interim file) for orientation (*), for annual hours corresponding to $ bin
annualR_tot{*}.ill	Illuminance (interim file) using DDS logic for orientation (*), for annual hours corresponding to $ bin
arad_{*}{#}_{$}.csv	3-channel spectral irradiance segment corresponding to the spectral bin (#, among 27 bins), for a given direction (*), for annual hours with sky spectra within a specific ($) CCT-bin
AnnRad_{*}{#}.csv	3-channel segments of spectral irradiance in a direction (*) for the spectral bin (#), combined using the indexing file for all annual hours

**Table 20:** Files merged from segmented spectral irradiance data from div{#} into the parent subfolder {main}\{case}\Spct.

File	Description
mrgSpect_n{*}.csv	81-channel spectral irradiance in a direction (*) for all annual hours
Mel_{*}.csv	Melanopic illuminance in direction (*) for all annual hours, using the spectral irradiance data factored with the CIEs026 melanopic function
Pht_{*}.csv	Photopic illuminance in direction (*) for all annual hours, using the v-lambda function
mEDI_{^}_graph.png	Annual hourly (365 × 24) MEDI plot for each direction
cie1931plt_A_graph.png	plotting spectral irradiance as CIE x/y coordinates, for a user-defined hour, for a given direction, on the CIE 1931 plot

**Table 21:** Supplementary _Data/Folder to be placed in C:\OWL\annuowl directory. Contains additional files necessary for running live simulation (e.g., weather file, spectral data, luminaire data).

File	Description
**aux-data\**	Folder containing additional material.
CIE1931.png	CIE xy chromaticity diagram.
reinsrc.cal	Radiance calculation file to compute Reinhart sky directions from bin number.
**eleclight\**	Folder containing luminaire data.
2722–001_98W.ies	Luminaire IES *.ies photometric data file for luminaire in office model.
42188062_(STD_LEO).IES	Luminaire IES *.ies photometric data file for luminaire in hall model.
linear_plot.png	Luminous intensity distribution, linear plot.
polar_plot.png	Luminous intensity distribution, polar plot.
**weather\**	Folder containing reference weather data.
AUT_Innsbruck.111200_IWEC.aowl	Precalculated spectral OWL weather file generated from EPW weather data input for location Innsbruck, Austria.
DEU_Stuttgart.107380_IWEC.aowl	Precalculated spectral OWL weather file generated from EPW weather data input for location Stuttgart, Germany.
Download_EnergyPlus_AUT_Innsbruck.epw.url	Download link for Energy Plus weather data file for location Innsbruck, Austria.
Download_EnergyPlus_DEU_Stuttgart.epw.url	Download link for Energy Plus weather data file for location Stuttgart, Germany.

## 4 Experimental Design, Materials and Methods

### 4.1 Generation of data in folder ./Models/

#### 4.1.1 Office model

For the Office model design, room 518 located in the building Technikerstrasse 13 of the University of Innsbruck, Austria, at 47.2640926° N, 11.3428399° E (https://maps.app.goo.gl/3NTJWAfMoYfYJZk18), was used as a template.

##### 4.1.1.1 Geometry

**Fig. 1:**
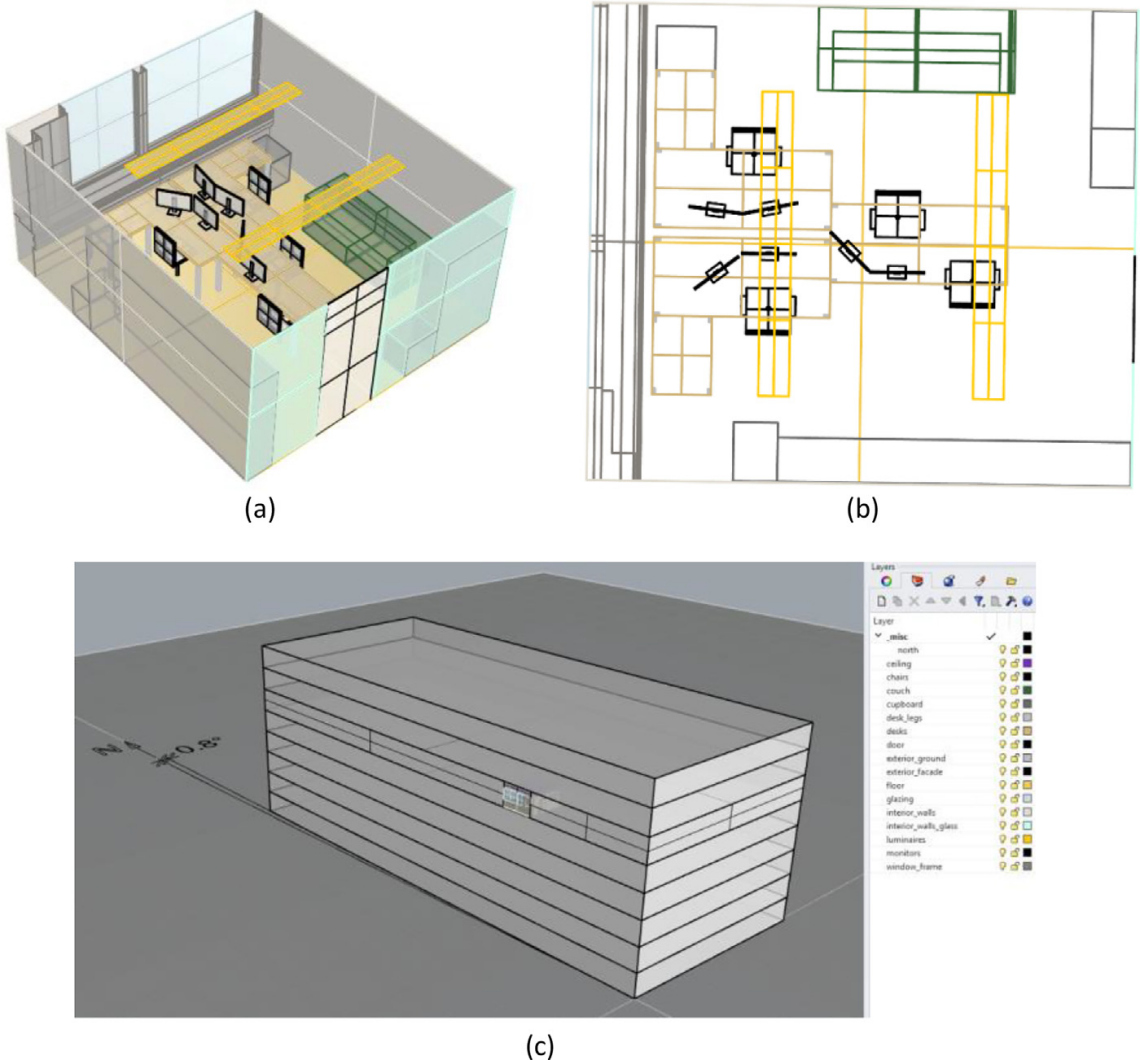
Visualization of the 3D model of the Office model: (a) overview and (b) plan of the reference room, (c) location of the room within the schematic whole building, with satellite image of surroundings mapped on the ground.

Modelling of the space and the furniture was based on measurements taken in the real office room. The 3D model of the office room was built in Rhinoceros 3D and is provided in the Rhino *.3dm format (*T70_Office/geometry/t70_reference_office_lab518.3dm*), see Fig. 1. The geometric characteristics and orientation of the office room are:

• 5.55 m x 4.86 m x 2.69 m sidelit office.
• Two windows (2.34 m x 1.66 m and 1.39 m x 1.66 m).
• Façade orientation: West (270.8°).

The shading lines describing the far and near horizon lines, and a total horizon line, were retrieved from the federal geoinformation online service TirisMaps [9]. Shading lines were retrieved for the location directly west of the building (representing the west façade) and at a height of 15 m above the exterior ground (*T70_Office/geometry/lab518_shading_lines.txt*).

##### 4.1.1.2 Weather data

To perform annual daylight simulations, weather data are required to describe the exterior situation. The nearest weather station to the building site is Innsbruck airport at a distance of about 0.8 km. We downloaded the IWEC weather data file in EPW format from the EnergyPlus website [10] (Download link provided in: *T70_Office/weatherdata/Download_EnergyPlus_AUT_Innsbruck.EPW.url*).

##### 4.1.1.3 Luminaires

**Fig. 2:**
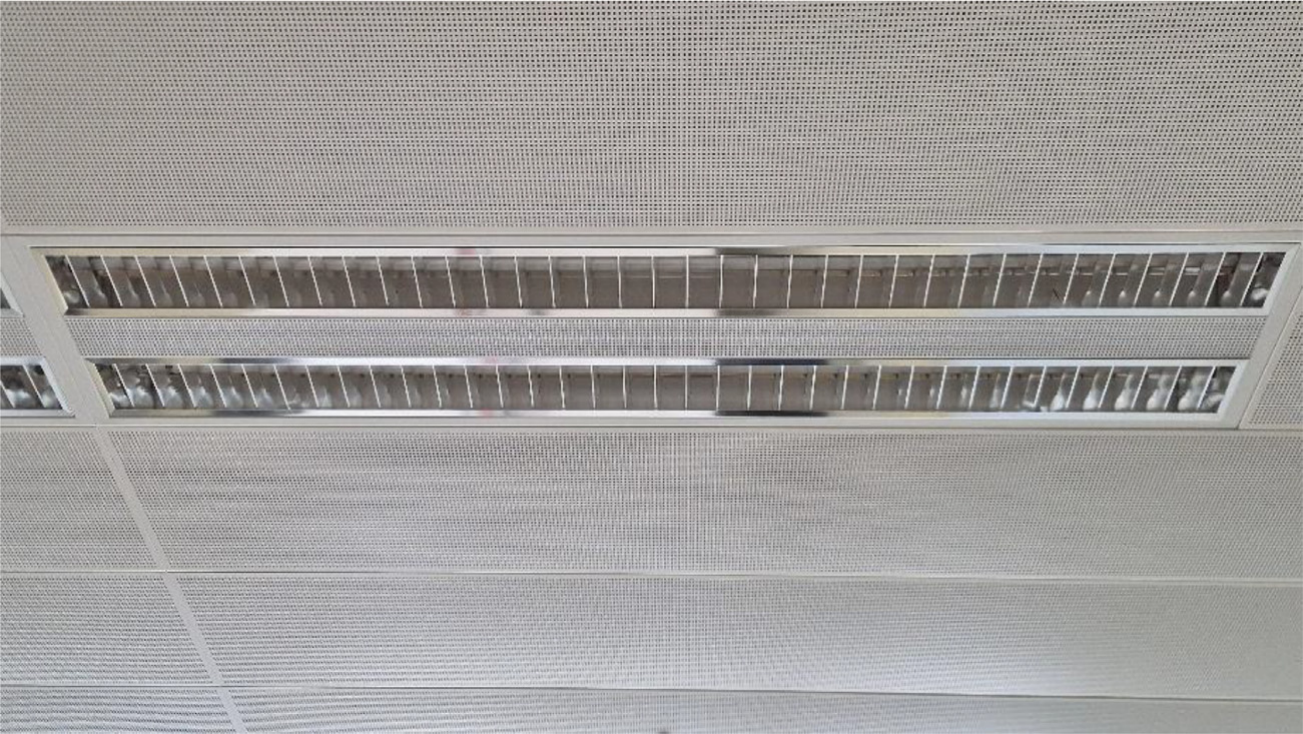
Recessed luminaire with fluorescent lamp in room 518.

Luminaires with fluorescent lamps are installed in room 518. The product specification from the manufacturer was available for the luminaire (Fig. 2), yet without data file. The luminous intensity distribution (LID) was modelled in the two sections from the PDF data sheet and the full spatial distribution was determined by interpolation.

Contrary to the original specification, each of the four luminaires in the office is equipped with two high output T5 lamps of type 840 with 49 W each. The system, thus, has 98 W with a total luminous flux of 8620 lm.

• 98 W (2 × 49 W).
• 8620 lm (2 × 4310 lm).
• Luminaire efficiency: 80.6%.
• Lower hemisphere: 100%.
• 4000K.
• Ra 80.

**Fig. 3:**
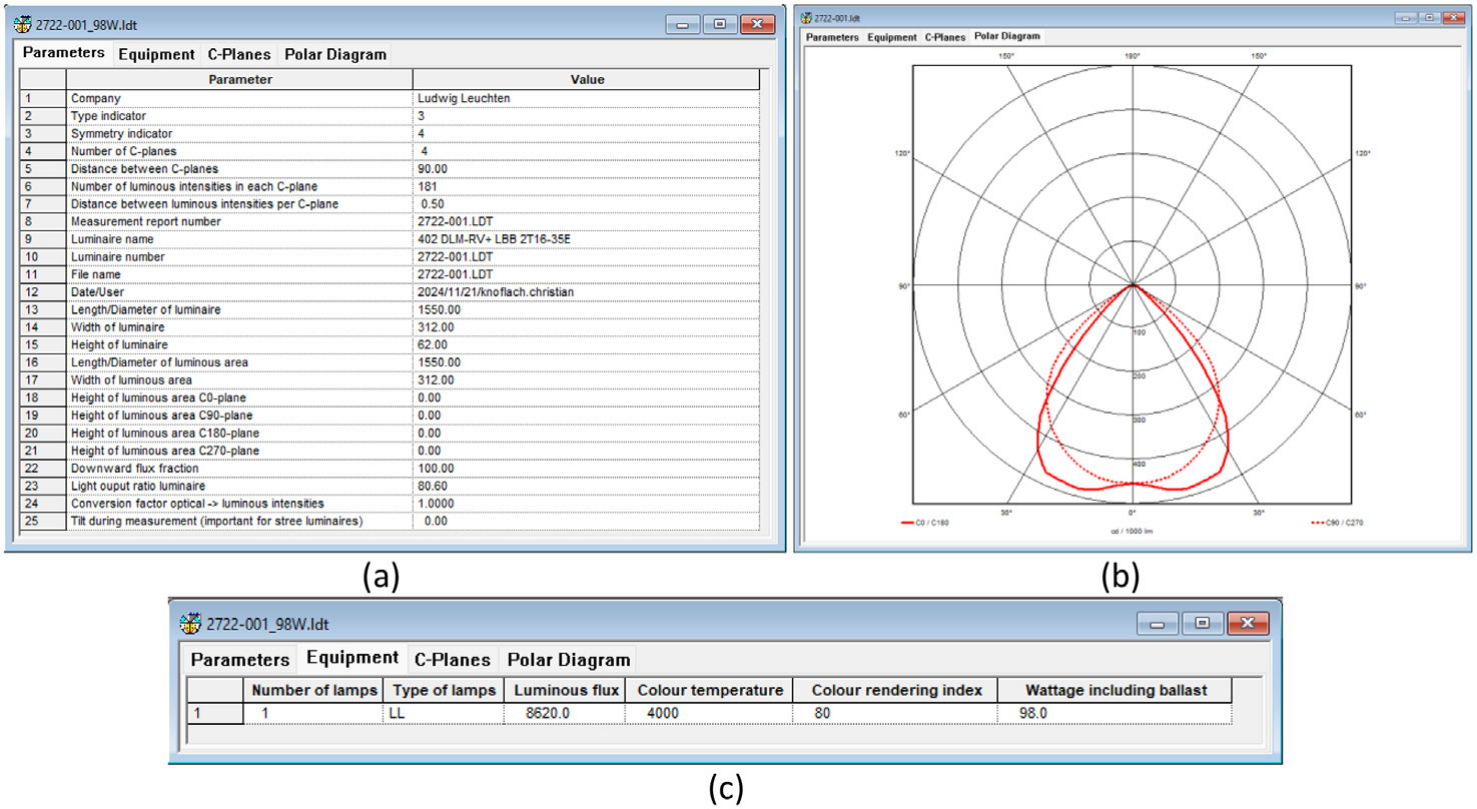
Luminaire specification in Eulumdat LDT file *T70_Office/luminaire/2722–001_98W.ldt:* (a) **l**uminaire parameters, (b) LID polar diagram, (c) luminaire equipment.

The LID was provided with the above information in the Eulumdat LDT file *T70_Office/luminaire/2722–001_98W.ldt*, see Fig. 3.

##### 4.1.1.4 Spectra

For the spectral distribution, a typical 840 fluorescent lamp spectrum is provided (*T70_Office/spectra/Fluorescent_840.csv*), see Table 4.

For the interior opaque materials, the spectral characteristics were measured with a handheld spectrometer (GretagMacbeth Spectrolino) in the real office room. The device measures spectral reflectances in the wavelength range comprised between 380 nm and 730 nm at 10 nm intervals using a tungsten-halogen light source. The measurements were performed with the “null” filter, i.e., not filtering the spectrum of the built-in source. The measurements include the total reflected light, including the specular component. For homogeneous surfaces (walls, door, desk, desk legs, chairs, monitor, cupboard, frame, ceiling base material), three measurements were taken for each, and the measurement with the median result for V(λ) was used. The deviations were below ±3% in absolute terms and – except for the black surfaces of the chairs and monitors – below ±10% in relative terms for all surfaces. For the perforated acoustic ceiling, the spectrum of the white base material was scaled in proportion to the material’s surface area (percentage of holes). The surfaces, which were not perfectly uniform in colour (floor, couch), were each measured six times. Each single measured spectrum was checked for plausibility to rule out errors, and then the six spectra were averaged. The data measured with the spectrometer are provided as *T70_Office/spectra/T70_office_{ceiling, chairs, couch, cupboard, desk, desk_legs, door, floor, frame, monitors, walls}.csv*, see Table 3*.*

Suitable spectral data for the geometry “Façade” and “Exterior ground” were selected from the Spectral Materials Database [11,12]. From the downloaded data (*T70_Office/spectra/{00027, 00769}_spectral.csv*), the “sci” (specular component included) was extracted manually by removing the column “sce” (specular component excluded) in a text editor and provided separately as *T70_Office/spectra/{00027, 00769}_spectral_sci.csv*.

The spectra of the transparent materials were generated using LBNL’s Optics [13] software based on spectral data from the International Glazing Database IGDB. From the *.usr files, which is the full output, the visual transmittance was copied to a separate *.csv file. Both files are provided in the *T70_Office/spectra/* folder for the insulating glazing unit *BauIng_518_Window{.usr, _t_vis.csv}* and the interior single pane glass wall *InterPane_Float_6mm{.usr, _t_vis.csv}*, see Table 2.

##### 4.1.1.5 Window

**Fig. 4:**
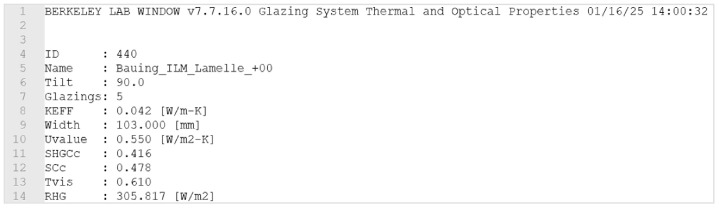
Extract from the WINDOW software report.

The transparent part of the west façade is equipped with two closed cavity windows, each of which consists of an internal triple pane thermal insulation glazing, a venetian blind system in the cavity, and an external single-pane glass. The glazing system was reconstructed in LBNL’s WINDOW software [14] and the performance data were determined. The full specification of the setup is provided in the output file *T70_Office/window/BauIng_Fensteraufbau.txt* (Fig. 4).

**Fig. 5:**
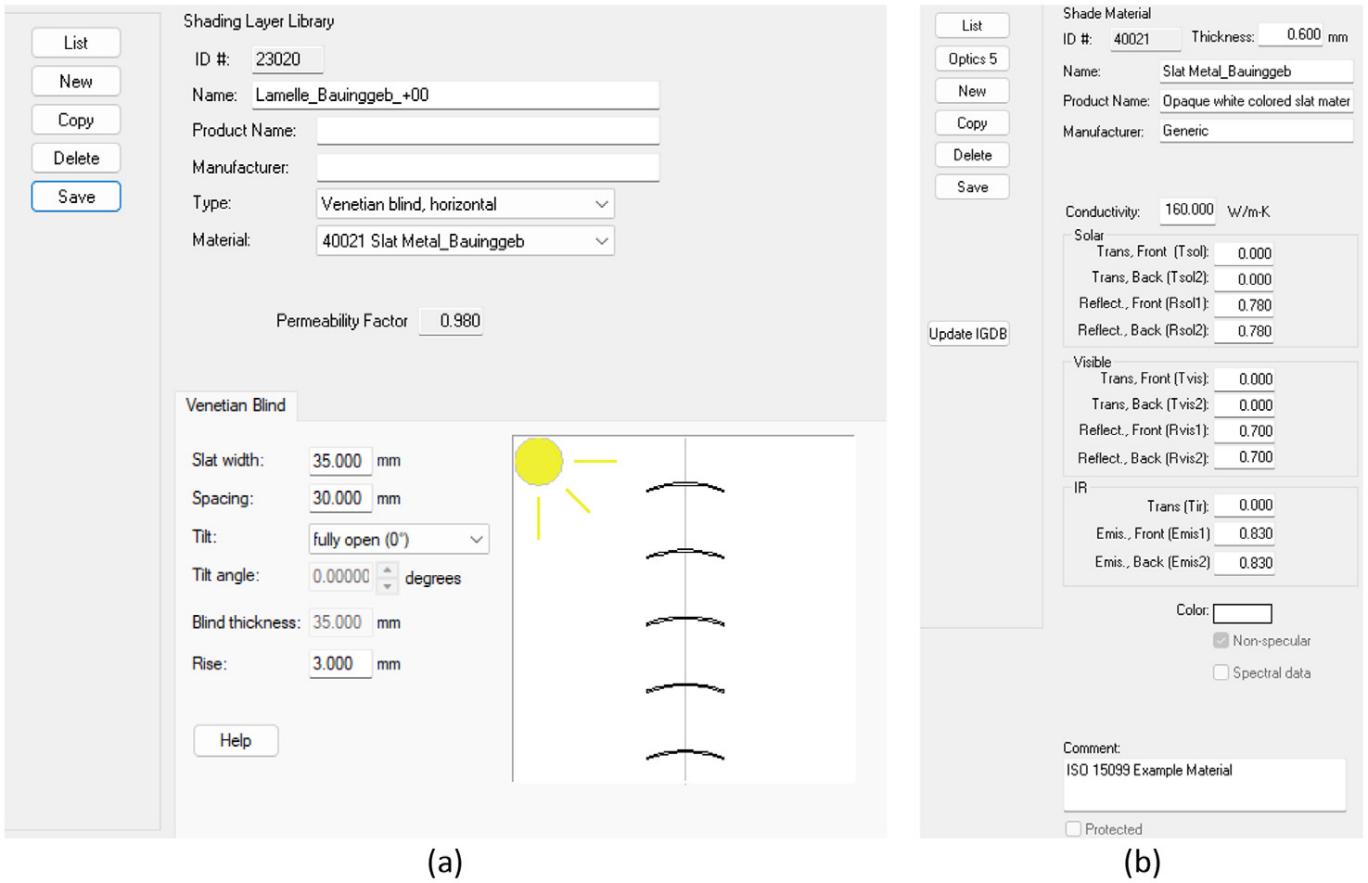
Definition of the venetian blind system in the reference office space in the WINDOW software: (a) Specification of the geometry, (b) specification of base material.

The venetian blind system was built in WINDOW in the shading layer library. Fig. 5 shows the specification of the base material as well as the definition of the geometry of the venetian blind system in the UIBK reference office space.

**Fig. 6:**
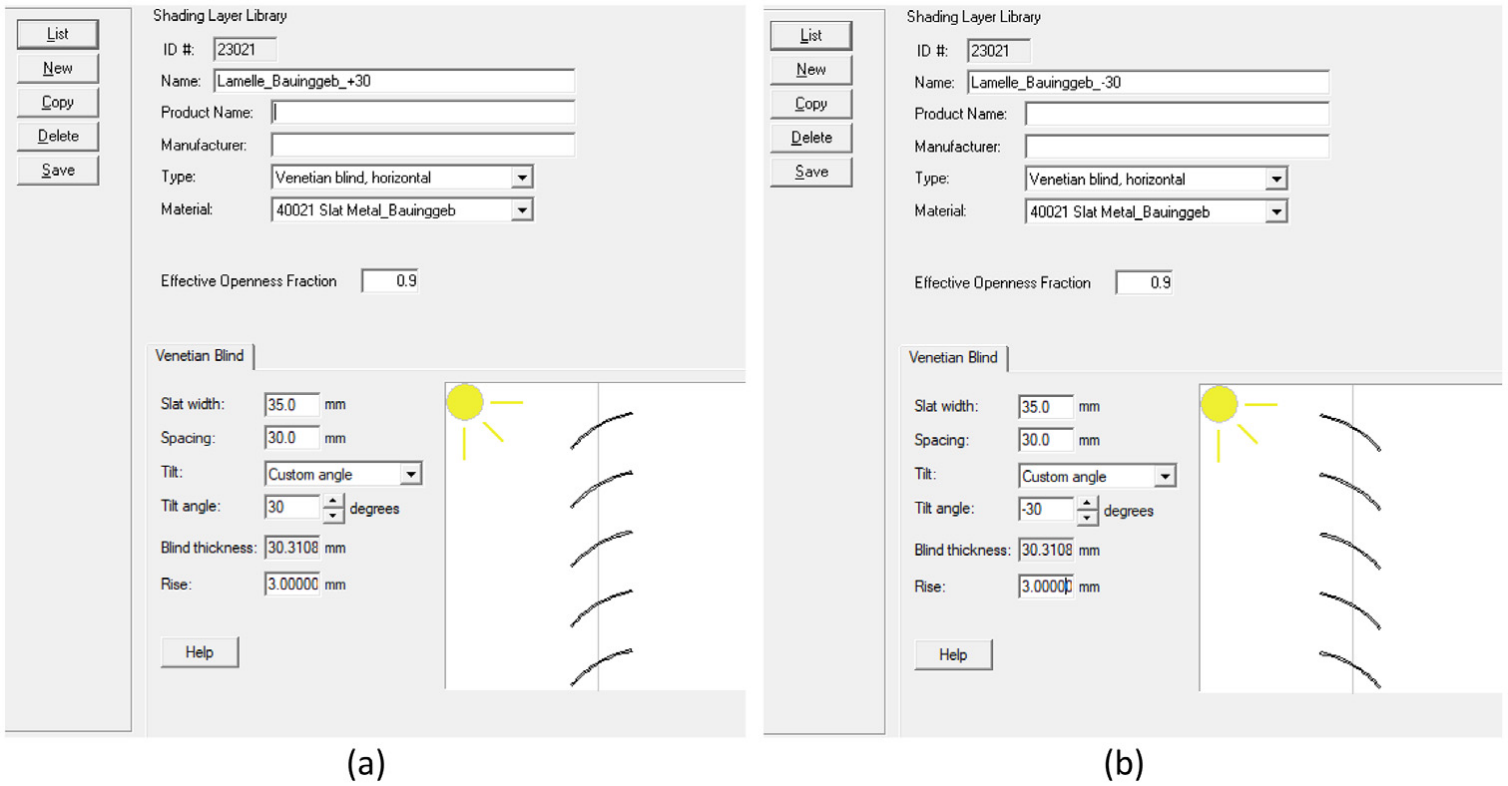
Example specification of the venetian blind system in the reference office space for non-horizontal settings in the WINDOW software: (a) Slats tilt angle +30°, (b) slats tilt angle −30°

The angular specification of the transparent façade is provided through the output from the WINDOW software. Since the focus was on lighting simulation, the modelling includes the glazing setup without frame, i.e., it reports the results for centre of glass, but omits thermal modelling of the overall closed cavity window. All data are provided for the glazing layers only (XXX) and for glazing layers plus venetian blind system in tilt angles from −85° to +85° in steps of 5° The orientation of positive and negative tilt angles is shown in Fig. 6. Based on these setting, the BSDF files were generated in XML and CSV file formats (T70_Office/window/BSDF/*.xml and T70_Office/window/CSV/*.csv), see Table 1.

#### 4.1.2 Hall model

The technical report CEN/TR 15193–2 [15] accompanies the lighting standard EN 15193–1:2017 and provides explanation and examples for implementing the standard. In Annex J, the report provides calculation examples for various building types. We modelled a reference factory hall based on “Example 1 – New design manufacturing building” specified in Section J.1.1.

##### 4.1.2.1 Geometry

**Fig. 7:**
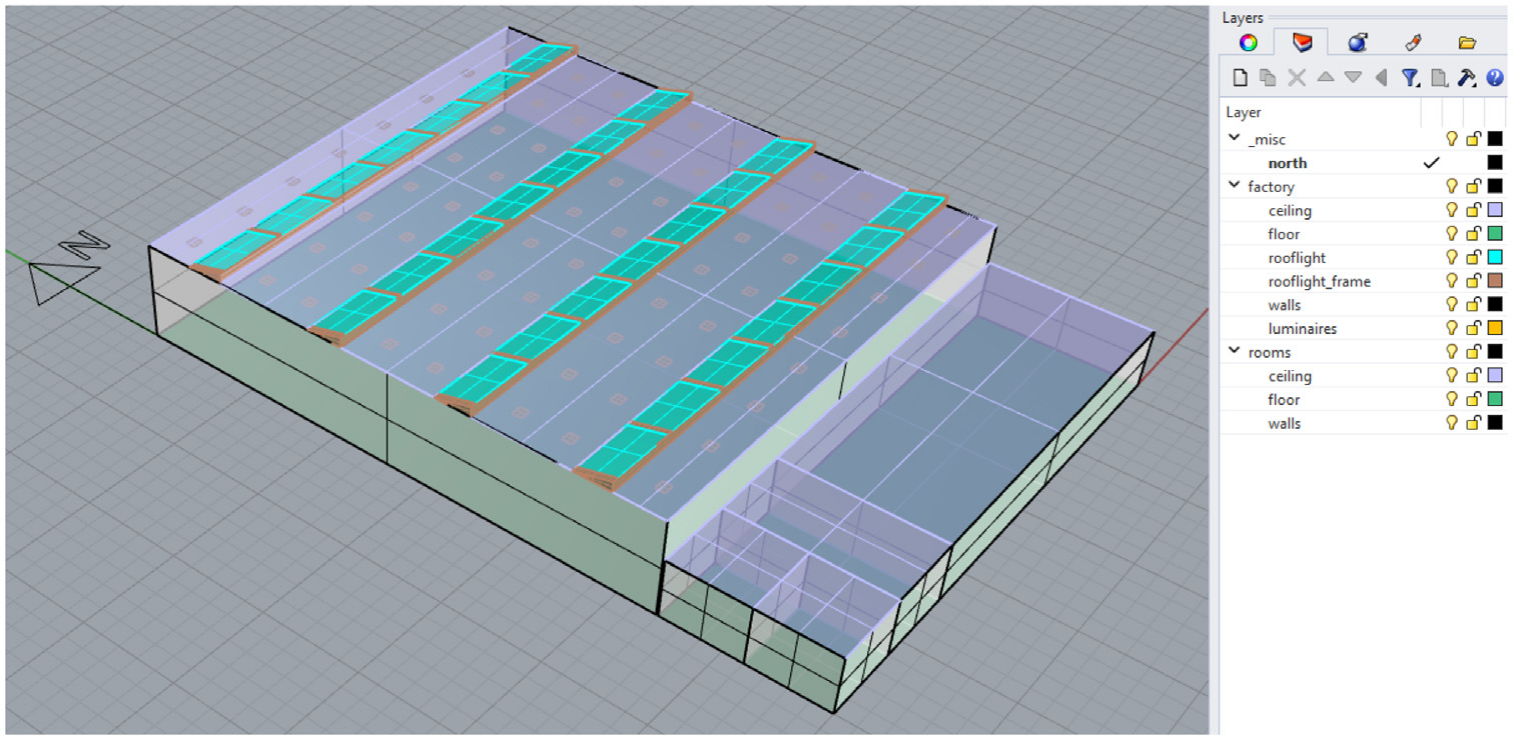
Visualization of the 3D model of the Hall model.

The 3D model of the factory hall was built in Rhinoceros 3D similar to the specification in CEN/TR 15193–2 and is provided in the Rhino *.3dm format (*T70_Hall/geometry/ t70_reference_hall.3dm*), see Fig. 7. The hall size and orientation are:

• 28 m x 26 m x 5 m overall dimensions of the hall.
• Rooflight orientation: North.
• External shading: none.

We aligned the skylights with the short side of the building and placed the building so that the rooflights are oriented towards the north.

##### 4.1.2.2 Weather data

The location of the virtual reference model was selected as Stuttgart, Germany. For the annual simulation, the IWEC weather data for Stuttgart was selected. The corresponding EPW file was downloaded from the EnergyPlus website [10] (download-link provided in: *T70_Hall/weatherdata/Download_EnergyPlus_DEU_Stuttgart.EPW.url*).

##### 4.1.2.3 Luminaire

**Fig. 8:**
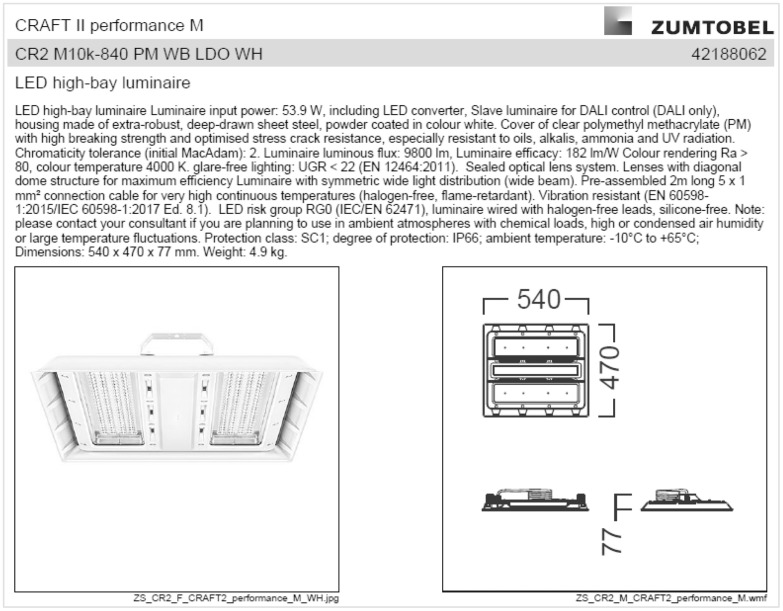
Luminaire specification and product image (source: Zumtobel Lighting [16]).

CEN/TR 15193–2 [15] specifies a daylight dependent dimmable LED luminaire for the factory hall. We performed an online search, and selected a typical high-bay luminaire as representative example for the reference hall (Fig. 8).

The luminous intensity distribution and the product datasheet were downloaded from the manufacturer’s website [16]. The main specifications are:

• Luminaire input power: 53.9 W Power factor = 0.94.
• Luminaire luminous flux: 9800 lm.
• Luminaire efficacy: 182 lm/W.
• Colour Rendering Index min.: 80.
• Correlated colour temperature: 4000 Kelvin.

**Fig. 9:**
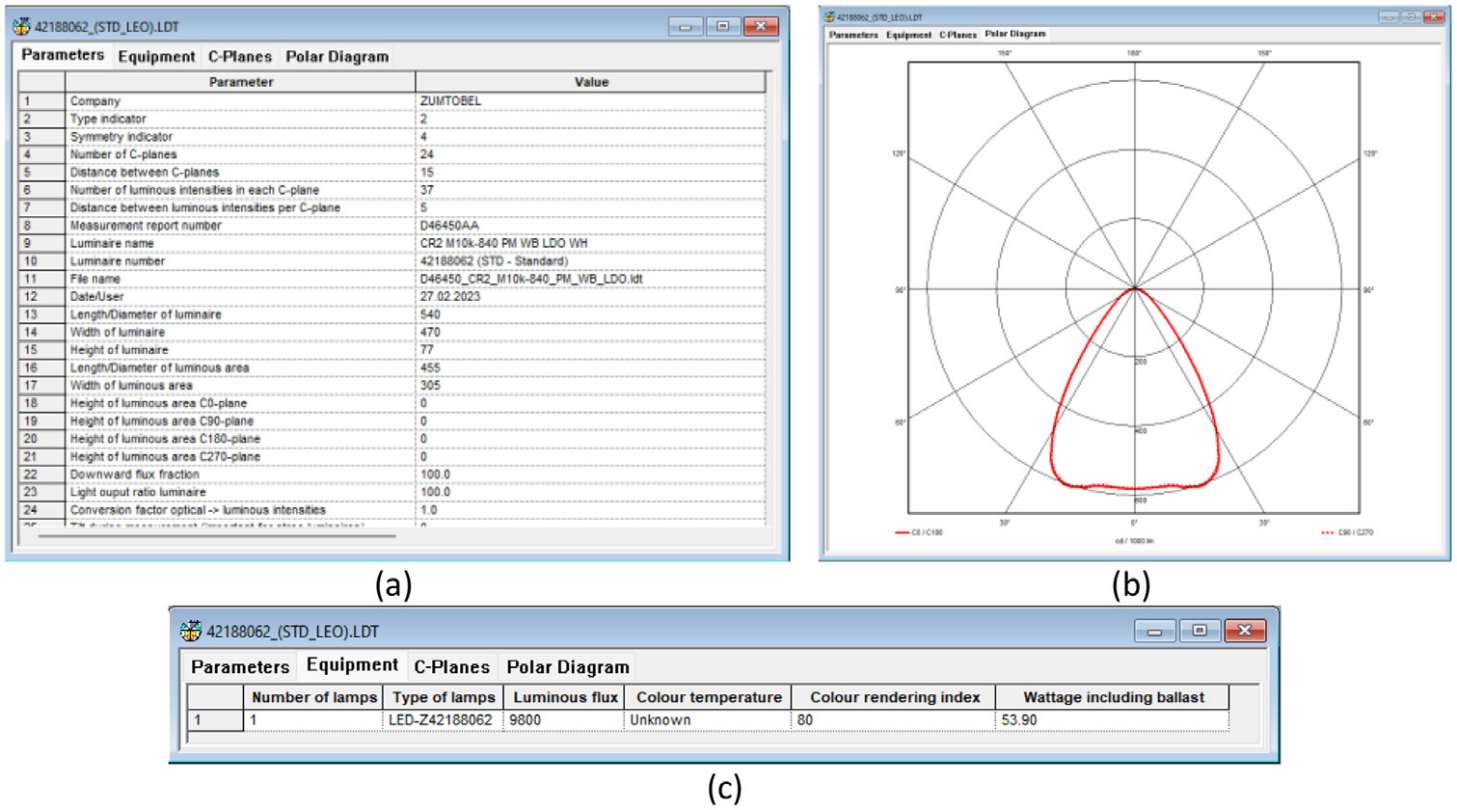
Luminaire specification in Eulumdat LDT file *T70_Hall/luminaire/42188062_(STD_LEO).LDT:* (a) Luminaire parameters, (b) LID polar diagram, (c) luminaire equipment.

The luminous intensity distribution data file in EULUMDAT format (*42188062_(STD_LEO).LDT*), see Fig. 9, and IES format (*42188062_(STD_LEO).IES*) as well as the datasheet are provided in the subfolder *T70_Hall/luminaire/.*

With this luminaire, we specified 8 rows with 8 luminaires each in the 3D geometry. Two rows of luminaires are mounted between the rooflight bands each, and one additional row each between the rooflight and the wall (see *T70_Hall/geometry/ t70_reference_hall.3dm*).

##### 4.1.2.4 Spectra

The typical spectrum of a 4000 K LED lamp is assumed. The spectral data for the CIE typical LED lamp 03 with 4103 K and Ra 85 was downloaded from the CIE website [17]. The provided spectral data was normalized to a maximum value of 1.0 and is provided as *T70_Hall/spectra/CIE_illum_LEDs_03_norm.csv*, see Table 8.

Suitable spectral data for the surface reflectances were selected from the Spectral Materials Database [11]. From the downloaded data (*T70_Hall/spectra/{00002, 00552, 00677, 00995}_spectral.csv*), the “sci” (specular component included) was extracted manually by removing the column “sce” (specular component excluded) in a text editor and provided separately as *T70_Hall/spectra/{00002, 00552, 00677, 00995}_spectral_sci.csv*, see Table 7.

The spectrum of the insulated glazing unit was generated using LBNL’s Optics software based on spectral data from the International Glazing Database IGDB. From the *.usr file, which is the full output, the visual transmittance was transferred into a *.csv file using a text editor. Both files are provided in the *T70_Hall/spectra/* folder as *Guardian_ClimaGuard_doublePane{.usr, _t_vis.csv}*, see Table 6.

**Fig. 10:**
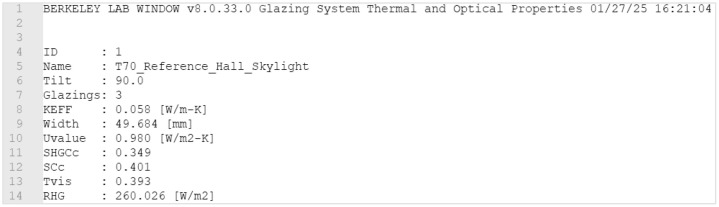
Extract from the report from the WINDOW software for the rooflight window setup.

The spectral transmittance of the translucent panel was modelled as generic 60% transmittance over all wavelength in the spectral range. The spectrum is provided in *T70_Hall/spectra/diffusing_shade_generic_60.csv. In the WINDOW software, this was included through specifying the solar and visible transmittance at 0.6 each (see* Fig. 10*).*

##### 4.1.2.5 Rooflight

For the angular specification of the rooflight window, the overall system consisting of a double pane insulation glazing with an additional third diffusing pane on the exterior was modelled in LBNL’s WINDOW software and the performance data were determined (Fig. 10). The full output is provided as *T70_Hall/rooflight/T70_Reference_Hall_Skylight.txt.*

**Fig. 11:**
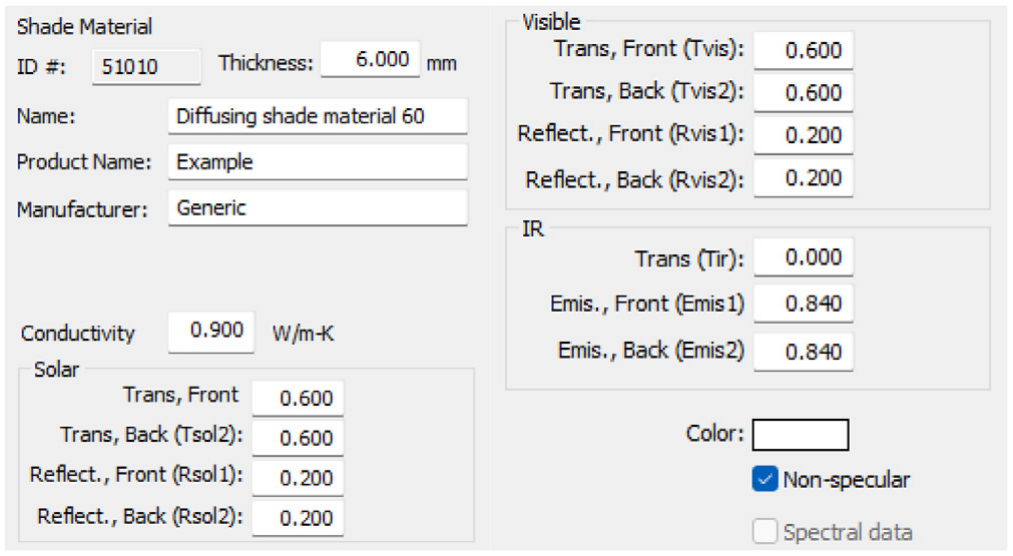
Specification of the diffusing pane characteristics for the rooflight window in the WINDOW software.

The diffusing pane was adapted from the Complex Glazing Database (CGDB) entry ID 31,002 and defined as a diffusing material with assumed spectrally neutral behaviour (Fig. 11).

The angular specification of the rooflight window (IGU plus the translucent panel) is provided through the output from the WINDOW software. Since the focus was on lighting simulation, the modelling includes the glazing setup without frame, i.e., it reports the results for the centre of the glass, but it omits thermal modelling. In WINDOW, the BSDF files were generated in XML and CSV file formats (*T70_Hall/rooflight/T70_Reference_Hall_Skylight.xml* and *T70_Hall/rooflight/ T70_Reference_Hall_Skylight.csv)*, see Table 5.

### 4.2 Generation of data in folder ./Implementation_Radiance/

The generation of the files is described below. For instances where the workflows for the office and the hall models required different steps, these are separately mentioned. The example implementation of the reference models and the simulations were performed using Radiance 6.1a, head release version February 3, 2026.

#### 4.2.1 Conversion of the models

##### 4.2.1.1 Preparation of geometry

The geometry in the provided 3D Rhino files was exported one layer at a time into separate OBJ files by selecting all objects on the respective layer selected and using “File – Export Selected” with filetype OBJ (files *.obj in *./office/objs*/ and *./hall/objs/*). Each of these files was then converted into a Radiance scene file *.rad using the Radiance tool *obj2rad*. For example, the floor of the hall model is converted with

*obj2rad -f ./hall/objs/factory_floor.obj > ./hall/Objects/factory_floor.rad*

The resulting Radiance scene files had generic modifier names (i.e., material assignments). These were manually updated using find and replace in a text editor to assign the appropriate material modifier names. All *.rad files were merged together with the *materials/materials.mat* description into *base_model.rad* (see below) to summarize the whole scene in one file. A second merged scene description was generated describing the scene with the electric lighting switched on (*base_model_night.rad*).

##### 4.2.1.2 Material specification

For the material definitions, the spectral data in the *.csv files were converted into Radiance spectral material descriptions using the PyRadiance [18] function *load_material_smd()*. For the example of the floor in the hall model, this is done with.

*pyradiance.load_material_smd("00002_spectral.csv*"*)*

The resulting Radiance material specification was then copied into the common material file for the respective scene (*./hall/materials/materials.mat* and *./office/materials/materials.mat*). If spectra are specified across different ranges in Radiance, internal resampling is performed, whereby the intersection of the defined output (i.e., calculation) spectral range and provided input (i.e., measured) spectral range for each material interaction is taken into account. For the measurement data here, values at wavelengths of 730 nm and above are assumed to be zero. As photometric and melanopic analyses are carried out, this is acceptable, since both weighting functions V(λ) and M(λ) are virtually zero above 730 nm. Should it be important for specific applications, the user can always prepare the spectral data accordingly in advance (for example, by extrapolation).

The emission spectra of the electric light sources, *./hall/spectra/CIE_illum_LEDs_03_norm.csv* and *./office/spectra/Fluorescent_840.csv*, were scaled to a luminous flux of an equal-energy, constant 1 spectrum, i.e., the integral over V(λ) (*CIE_xyz_1931_2deg.csv* downloaded from [19]), to not affect the photopic Y measurements of the original IES files *hall/sources/42,188,062_(STD_LEO).IES* and *office/sources/2722–001_98W.ies*. To generate the normalized spectra, which can be used as multiplier (*hall/spectra/Guardian_smult_780–380.txt* and *office/spectra/ Fluorescent_840_smult_380–780_81.txt*), the tools *tabfunc* and *rcalc* are used. These spectra were then copied in a text editor to *materials/materials.mat*.

The photometric properties of the office luminaire were previously converted from the Eulumdat format *(2722–001_98W.ldt)* into the IES format using a free online converter [20]. The luminaire IES files were converted to Radiance input files *.rad using the tool *ies2rad.*

The V(λ)-normalized transmission spectrum for the window glazing was multiplied against the BSDF XML files using the *rmtxop* tool to obtain spectral BSDFs. To generate the normalized spectra, which can be used as multiplier (*hall/spectra/Guardian_smult_780–380.txt* and *office/spectra/BauIng_518_smult_780–380.txt*), the tools *tabfunc* and *rcalc* are used. For example, for the hall rooflight, the scaling of the spectra is then done with

*rmtxop -ff -c `rcalc -e '$1=0;$2=$1;$3=0′ spectra/Guardian_smult_780–380.txt` rooflight/T70_Reference_Hall_Skylight.xml > mtx/SpecSkylight.mtx*

(see *_{hall,office}_step01.sh*).

#### 4.2.2 Workflow to perform spectral annual daylight simulations

##### 4.2.2.1 Generation of sky and daylight matrices

The climate data files were converted to files including daytime-hours (8 to 18) only using the *sed* tool (resulting files: weatherdata/*day.epw). Using these weather data files, spectral sky matrices were created with the tool *gensdaymtx* for a sky subdivision according to Reinhart MF:4. To prepare the later calculation of overall daylighting metrics as well as solar-only evaluations, separate matrices were created (*_daytime_sky4.mtx and *sunonly_sky4.mtx). This also creates the directory *atmos_data* and the including files. The program calls are included in *_{hall,office}_step02.sh*.

Next, the daylight contribution matrix D connecting incident window directions to sky patches was generated using the *rxfluxmtx* tool. To act as light source in the calculation, the window or rooflight geometry and materials were adapted. For the window in the office model, the Radiance geometry from *objects/glazing.rad* was copied in a text editor to *window/window_kf.rad* and supplemented with a header that triggers the use of Klems resolution. The uniform *light* source *window_light* was defined and applied to the window geometry. For the rooflight in the hall model, the *window/roof_skylight.rad* was prepared with the header defining the Klems resolution and a uniform *glow* source named *skylight_mat*. To emit light on the correct side (into the hall), the geometry of the rooflight windows was then inverted and the defined material *skylight_mat* applied using the following xform command:

*xform -I -m skylight_mat objects/factory_roof_light.rad >> window/roof_skylight.rad*

For the calculation of the daylight matrix, the required sky receiver was manually created in a text editor with both a uniform ground and a Reinhart MF:4 sky subdivision (*sources/sky_r4.rad*). The *rxfluxmtx* program for the generation of the daylight matrix *mtx/daylight_r4.mtx* is given in *_{hall,office}_step03.sh*.

The resulting daylight matrix was then multiplied with the daytime-hours sky matrix (resulting in mtx/*_daytime_kf.mtx) and further with the respective BSDF files to obtain matrices including the overall light propagation between the sky and the exiting patches of the window/skylight represented through its BSDF (files mtx/*_day_{blinds, noblinds, skylight}.mtx). The matrix multiplication calls using *rmtxop* are included in *_{hall,office}_step04.sh*.

##### 4.2.2.2 Generation of view matrices

The image view matrix for each of the four cardinal hemispherical fisheye views from the center of the hall, and for the three sitting positions in the office, were calculated using the *rfluxmtx* tool called from *_{hall,office}_step05.sh*. The definitions of the views in the hall were manually written in a text editor and saved in *views/h{east,north,south,west}.vf*. For the office model, the view definitions were included in the *_office_step05.sh* script.

For the calculation of illuminance values, workplane sensors were generated. For the office model, a point cloud was generated in Rhino3D and exported as points file *pts/workplane.pts*. In each line, “0 0 1″ was added in a text editor to describe the up-vector, i.e., the orientation of the sensor. For the hall, the file *pts/workplane.pts* was created using

*cnt 28 26 | rcalc -e '$1=$2+0.5;$2=$1+8.6;$3=0.75;$4=0;$5=0;$6=1′ > pts/workplane.pts.*

The calculation of the view matrix V for illuminance readings on the workplanes was again performed using the tool *rfluxmtx*, now with the option *-I* to switch to irradiance calculations (*_{hall,office}_step06.sh*). Further, the direct-only view matrices for illuminance values on the workplane were generated (*rfluxmtx* with the options *-I -ab 1*, called in *_{hall,office}_step07.sh*).

##### 4.2.2.3 Calculation of annual daylight results

For the illuminance calculations, the calculated matrices were multiplied using *rmtxop* to obtain annual results for the daytime hours 8–18, resulting in the matrices *hall/mtx/Stuttgart_day_wp_ill.mtx* and *office/mtx/Innsbruck_day_{blinds,noblinds}_wp_ill.mtx*. The tools *rcollate* and *rcomb* were then used to compute annual daylighting metrics, such as sDA or UDI (*_{hall,office}_step08.sh*). Similar, the direct sun-only contribution was calculated (*hall/mtx/Stuttgart_sunonly_wp_direct_ill.mtx* and *office/mtx/Innsbruck_sunonly_{blinds,noblinds}_wp_direct_ill.mtx*), and used to compute the ASE metric in *_{hall,office}_step09.sh*).

The image view matrices were combined using the *pvsum* tool to generate hourly images for the single viewpoints (*_{hall,office}_step10.sh*). Each of these images was then evaluated with *evalglare* to compute the DGP glare metric. Using *rcalc* on the results, the fraction of daylight hours with a DGP above a threshold of 0.4 was computed (*_{hall,office}_step11.sh*). The format definition *misc/glare.fmt was* manually created in a text editor to match the output of *evalglare*.

##### 4.2.2.4 Calculation of melanopic equivalent daylight illuminance

Calling *_{hall,office}_step12.sh*, hyperspectral images of night-time views (electric lighting only) were computed using the *rxpiece* tool with the parameter *-cs 20* to specify 20 spectral bands to be used. Next, the value mEDI was computed using the tools *rcomb* and *rcalc* by dividing the vertical melanopic lux by the equivalence factor for daylight (1.10). Finally, the hyperspectral images result/*.hsr were converted to HDR images results/*.hdr using the tool *ra_xyze* with option *-r*, and further with the *ra_tiff* tool to the TIF images results/*.tif for viewing.

##### 4.2.2.5 Calculation of electric lighting energy demand

To calculate the energy demand for electric lighting based on a daylight-dependent dimming control using a ceiling-mounted sensor, the following steps were performed. First, the workplane illuminance from electric lighting only was calculated using the *rtrace* tool with option *-I+*, resulting in the illuminance matrix *mtx/electric_wp_ill.mtx* (*_{hall,office}_step13.sh*). Similar, the ceiling sensor illuminance from electric lights only was computed (*_{hall,office}_step14.sh*). Using the tool *total* with option *-m* the average workplane illuminance with electric lighting at 100% was computed (*_{hall,office}_step15.sh*). In *_{hall,office}_step16.sh*, the relative daylight contributions per Klems direction at the ceiling sensor, i.e., a contribution vector, was calculated using the *rxfluxmtx* tool. Then, we used this vector in a combined *rmtxop* and *rcalc* call to determine the light levels to achieve a target illuminance of 127.2 lx at the ceiling sensor corresponding to an average illuminance of 625 lx on the workplane. From this, the number of occupied hours and the annual electric lighting energy demand were computed with the *rcalc* tool (*_{hall,office}_step17.sh*).

In the office model, additionally, the results for a controlled façade system were calculated. The position and direction of the sensor were determined from the Rhino3D model and saved in *office/pts/facade_sensor.pts*. For this sensor, a daylight coefficient vector for an exterior, facade-mounted, sensor was computed using the *xfluxmtx* tool (*_office_step18.sh*). Using the *rmtxop* tool, the annual exterior vertical irradiance readings for the façade-mounted sensor were calculated (*mtx/Innsbruck_daytime_facade_ext_irrad.txt*) and the and control setting based on an 120W/m^2^ threshold determined using *rcalc*, resulting in *mtx/Innsbruck_daytime_useblinds.txt* (*_office_step19.sh*).

Using *rlam* and *rcalc*, the controlled electric lighting levels were calculated (*mtx/electric_light_levels_control.txt*) and further processed to obtain operating hours and the annual energy demand (*_office_step20.sh*).

### 4.3 Generation of data in folder ./Implementation_OWL/

The Grasshopper scripts *DiB_OWL_Office.gh* (for the Office model) and *DiB_OWL_Hall.gh* (for the Hall model) provide the workflow to run simulations for these cases in OWL [7], and to replicate the dataset provided in the ZIP files. The pre-simulated dataset in these two folders can also be directly visualized using the additional Grasshopper scripts (respective *_Cached.gh). The visualized results can help a user get an overview of the workflow and evaluations, without the need to run the intensive simulations, and also serve as a training prior to simulating their own cases.

**Fig. 12:**
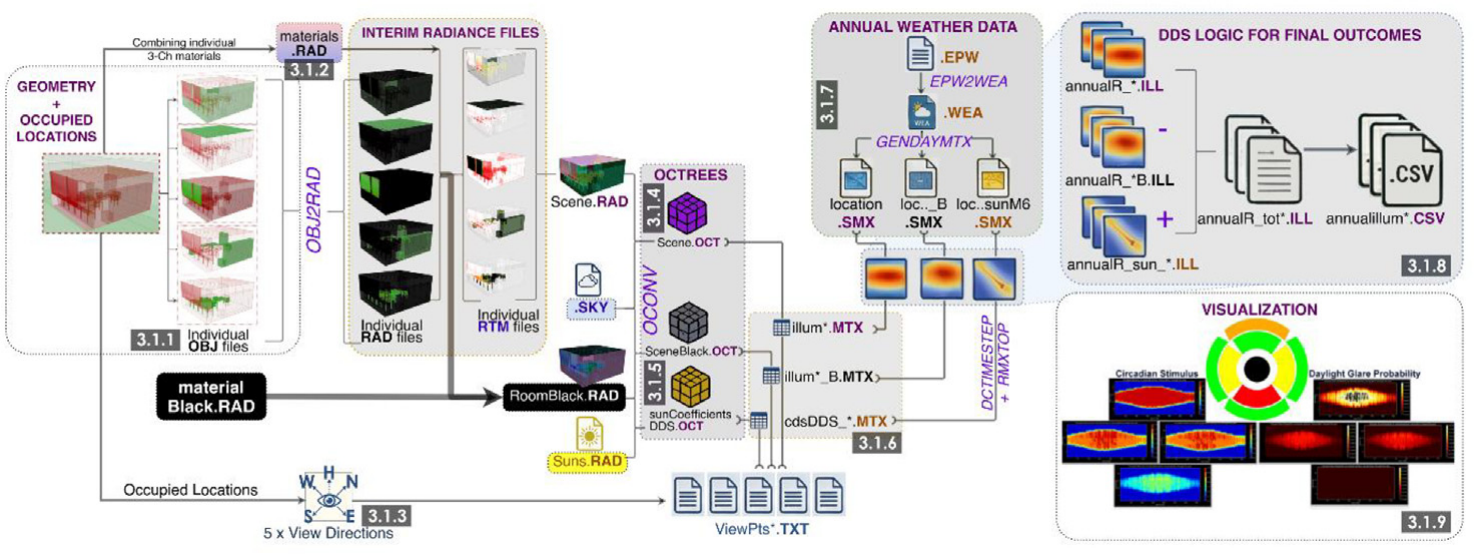
Workflow in OWL as described in section 4.3.1.

**Fig. 13:**
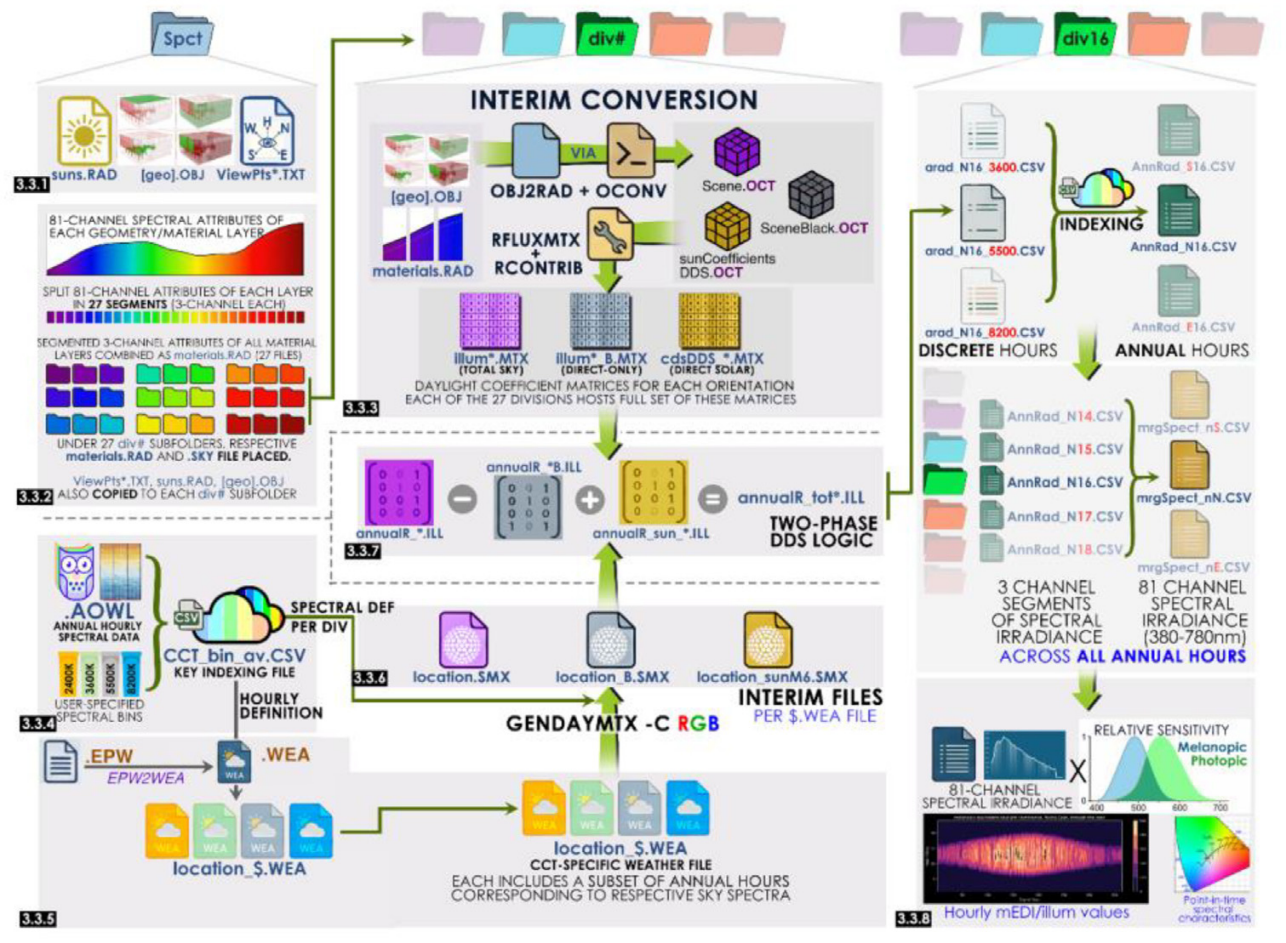
Detailed workflow for 81-channel spectral simulation in OWL across dynamic hourly spectral skies, as described in section 4.3.3.

The diagrams in Fig. 12 and Fig. 13 give a simplified overview of the lighting simulation workflows within OWL that were applied to generate the provided data. Among these, Fig. 12 demonstrates the generation of occupant-centred luminous metrics evaluated at head-height on a vertical plane, across four cardinal directions using a quicker workflow, which is as further detailed in section 4.3.1. The infographic in Fig. 12 can also be used to comprehend the workflow presented in section 4.3.2, where space-centred luminous metrics are evaluated on a horizontal plane. Fig. 13 presents a detailed multi-spectral simulation workflow that is used for evaluating non-image forming metrics, via an 81-channel spectral simulation across dynamic hourly spectral skies, which is detailed in section 4.3.3.

The example implementation of the reference models and the simulations were performed using OWL 3.2, version 01/2026.

#### 4.3.1 Workflow within the occupant-centred, OVNI-based evaluations

The OVNI (Occupant Visual and Non-visual Illumination) diagrams are the sombrero-like plots (that look like UFOs, which in French are termed *Objet volant non identifié*, or OVNI) that describe occupant-centred performance metrics related to human factors associated with daylight. The central hemisphere of the OVNIs denotes visual sufficiency, its first set of ring-segments across four orientations describes circadian autonomy, while its second set of ring-segments describe protection offered from discomfort due to glare. Each of these performance indicators are presented in four thresholds: “high” (green), “medium” (yellow), “minimum” (orange), or “non-compliant” (red). The following steps are followed in our Grasshopper script to evaluate OVNI-based occupant centred evaluations.

##### 4.3.1.1 Exporting obj files

From Grasshopper, the script exports the internalized geometry for different layers (based on material characteristics) as separate *.obj files, naming them based on the materials they are attributed.

##### 4.3.1.2 Generating sky and material attributes

A *.sky file is generated together with a *materials.rad* file, which sequentially includes the three-channel reflectance or transmittance values of individual materials (together with their specularity and roughness, if applicable).

##### 4.3.1.3 Exporting sensor points corresponding to observer’s positions and viewpoints

Five files called ViewPts*.txt – with * corresponding to the four cardinal directions (“N”, “E”, “S”, “W”), and a fifth point facing vertically upward (“H”) – are created. For each defined observer, these files collate the x, y, z coordinates of their position, together with three additional coordinates corresponding to their view vector – so six values per observer, in total, for each viewing direction.

##### 4.3.1.4 Interim conversions using Radiance utilities to generate scene octrees

Each *.obj file is individually converted to a *.rad file using the Radiance *obj2rad* tool and further meshed together with the material file *materials.rad* into separate *.rtm (Radiance triangle mesh) files. They are then combined into a *scene.rad* file, which together with the *.sky file is converted to a Radiance octree (via Radiance *oconv*) termed *scene*.oct.

##### 4.3.1.5 Creating other octrees for supporting two-phase direct sun calculations

For isolating the direct-only part of the sky, a black plastic material having zero reflectance for each channel is created, and saved in the *materialBlack.rad* file, while another interim file termed *roomBlack.rad* combines each layer's *.rad object (previously generated from *.obj files in step 3.1.4) with this black material. Using the Radiance tool *oconv*, another octree termed *sceneBlack.oct* is created that combines the *materialBlack.rad* and *materials.rad* files, together with the *roomBlack.rad* and with the *.sky file generated in step 3.1.2.

For supporting the direct-sun evaluations, a *suns.rad* file is created, setting the angular diameter of the sun at 0.533 degrees, i.e., as seen from Earth, having intensities for each channel at 1 million W/sr/m^2^, and with positions for the sun defined via their cartesian coordinates – using the Reinhart multi-factor (MF) subdivision at 5. Using *oconv*, and similar to the direct-only part (with the exception of using the *suns.rad* in place of the *.sky file), another octree termed *sunCoefficientsDDS*.oct is generated.

##### 4.3.1.6 Calculating daylight coefficient matrices for total sky, direct-only sky, and direct sun, for each orientation

To calculate diffuse sky coefficients, five illum*.mtx files are generated using the Radiance *rfluxmtx* and *rcontrib* utilities – with * corresponding to each of the five orientations by combining inputs from the ViewPts*.txt files described in step 3.1.3 – and sampling the 145 Tregenza patches in the sky vault (MF=1) via the *skyglow* and *groundglow* modifiers. Using the *reinhartb.cal* definition, the contributions from each patch to the sensors are recorded, with 5 ambient bounces and high sampling density, to produce a daylight coefficient matrix of 145 RGB radiance values, and saved as illum*.mtx files for each orientation. This matrix corresponds to the total-sky contributions for each defined orientation. Following the total-sky matrix, two additional sets of matrices are generated for the indirect and direct solar components. Among these, the illum*_B.mtx use the black-material scene to capture diffuse contributions from the sky at first bounce (-ab 1), while cdsDDS_*.mtx include the direct solar coefficients. For this direct-sun matrix, the *suns.rad* source is used to map contributions from each sun-position to the sensors, again using a single bounce (-ab 1). Effectively, three resulting types of daylight coefficient matrices (*.mtx) are created for each view vector defined in step 3.1.3., with total sky denoted as illum*, direct-only sky as illum*_B, and direct solar as cdsDDS_*.

##### 4.3.1.7 Generation of solar matrices to use with the three types of daylight coefficients

The EnergyPlus weather file *.epw corresponding to the location is first converted to a *.wea file via the Radiance *epw2wea* utility, and is denoted as the *location.wea* file. This wea format is a DAYSIM-style format that is required by the Radiance *gendaymtx* utility. The *.wea file is then used as input for three separate *gendaymtx* calls, resulting into the generation of a *location.smx* file, which includes the total sky radiance for 145 patches over 8760 hours, a *location_B.smx* file, which includes the direct-only sky component for the sky patches and used for subtracting the background, and a *location_sunM6.smx* file, which includes a high-resolution solar matrix generated for exact sun positioning.

##### 4.3.1.8 Combining inputs from solar matrices and daylight coefficient matrices using DDS logic

Annual hourly illuminance values are further derived through a multi-step matrix multiplication (and subtraction) process. First, the Radiance *dctimestep* utility is used for the total-sky files, to cross-reference the daylight coefficient matrices (illum*.mtx) with the hourly weather data (*location.smx*) to produce interim annualR_*.mtx files that contain total spectral contributions for each orientation. Simultaneously, the direct-only contributions are evaluated using the corresponding files (i.e., illum*_B.mtx and *location_B.smx*) to generate the direct-only spectral contribution within the interim annualR_*B.mtx files. The three-channel matrices are then processed via the Radiance *rmtxop* utility, using standard luminous efficacy weights of 47.4, 119.9, and 11.6 to convert RGB radiance into hourly illuminance (*.ill) files – for the total sky (i.e., annualR_*.ill), the direct-only contribution (i.e., annualR_*B.ill) and the direct-solar component (i.e., annualR_sun_*.ill).

Following the 2-Phase DDS logic, the final result is then evaluated for each hour by taking the total sky illuminance, subtracting the coarse direct-only component to prevent double-counting, and adding the high-resolution direct-solar illuminance for sharp and accurate shadows. These annual total illuminance values (saved as annualR_tot*.ill), together with the files generated for the interim steps, are all evaluated for the five orientations (*). After removing the header data in the *.ill files, the final result (i.e., annualR_tot*) files are then translated to *.csv files, and saved as annualillum*.csv These are saved separately, corresponding to each orientation specified in step 3.1.3., and with each file comprising 8760 rows (i.e., annual hourly values), or as many as specified in the *.epw file, respectively. The columns correspond to the number of evaluation points.

##### 4.3.1.9 Post processing from hourly illuminance values for each orientation

The vertical illuminance hourly values (from annillum*.csv) are used for further occupant-centric derivations. For calculating circadian autonomy, the hourly illuminance for each vertical orientation is factored with the precalculated CIE-z coordinate (from the aowl file corresponding to a location, as defined in the script) to generate the approximated circadian stimulus following Truong's approach [21]. This results in annual hourly CS values (saved in the ann_CS_*.csv files), which are further reduced to performance bins of “high”, “medium”, “minimum”, and “non-compliant” and stored in CSautonomy*.csv files. For evaluations of daylight glare, the vertical illuminance values for each hour are directly translated into simplified DGP (sDGP) values, which are then stored as ann_DGP_*.csv. With interim transpositions, these derived performance metrics are then mapped as an annual hourly (e.g., 365×24) grid, converted to *.png image files, and saved as CS_*_graph.PNG and DGP_*_graph.png for each vertical orientation *North, East, South*, and *West*.

#### 4.3.2 Workflow within the space-centered, horizontal grid-based evaluations

##### 4.3.2.1 Exporting the floor grid model and the sensor points on the horizontal grid

The floor surfaces identified for grid-based evaluation are exported as a 3D model (*floorgrid.3dm*). With a vertical offset corresponding to the user-defined or standard sensor placement height above the floor surface, together with user-inputs on the x- and y- separation between sensors, the three-dimensional coordinates are saved for each sensor point in a text file (*GrdPtsH.txt*). For each point, a vertically-upward facing direction vector (+Z, i.e., (0,0,1)) is defined in the text file.

##### 4.3.2.2 Creating three types of daylight coefficient matrices

Three octrees corresponding to total sky (*scene.oct*), direct-only sky (*sceneBlack.oct*), and direct-solar contribution (*sunCoefficientsDDS.oct*), are generated in interim steps in a similar fashion to steps 3.1.4. – 3.1.6. of the occupant-centred evaluations. These are further translated via the Radiance *rfluxmtx* and *rcontrib* utilities, while combining inputs from the sensor placement file (i.e., *GrdPtsH.txt*), to generate the three daylight coefficient matrices corresponding to the sensor placements on the horizontal grid. These matrices are saved as *ill_Hrz.mtx, ill_Hrz_B.mtx* and *cdsDDS_Hrz.mtx,* all for the vertically upward facing direction vector. This step only differs from its occupant-centred counterpart in step 3.1.6. in the use of the point-files used for evaluating daylight coefficients. While five ViewPts*.txt inputs are used in the occupant-centred evaluation, each corresponding to a different orientation (see 3.1.3.), here this step takes input only from the upward facing sensors on the horizontal grid (in *GrdPtsH*.txt) for evaluating the three categories of daylight coefficients for the two-faced DDS method, i.e., total-sky, direct-only part, and direct-solar component.

##### 4.3.2.3 Generating hourly illuminance values and deriving climate-based daylight metrics

In a process similar to steps 3.1.7. and 3.1.8. of the occupant-centred evaluations, the weather file (*.wea) and interim solar matrices (*.smx) are generated, and annual hourly evaluations for the grid-based sensors are evaluated to yield hourly-illuminance values. For total sky (*annualR_Hrz.ill*), direct-only component (*annualR_HrzB.ill*) and direct-solar component (*annualR_sun_Hrz.ill*) files, with the 2-phase DDS logic further followed to evaluate the annual hourly illumination over each grid point. This is saved within the *annualR_totHrz.ill* file, which is then converted into a *.csv format by removing the header (*annualillum_Hrz.csv*). This data is then used to evaluate daylight metrics, including daylight autonomy (DA), continuous daylight autonomy (cDA), useful daylight illuminance (UDI), average illuminance, annual sunlight exposure (ASE), and scores for meeting the minimum, medium, and high thresholds of spatial daylight autonomy. These metrics are evaluated for each sensor location, and for ease of visualization over the grid, are saved in another text-based file (*ann_metrics.csv*).

In parallel, for calculating an approximate Daylight Factor (DF) to be visualized over the grid together with other daylight metrics, a *skyCIEOvercast.rad* file is generated, which uses a source with uniform glow to represent a simplified overcast sky (note that this is not a true CIE overcast sky, which has a non-uniform 3:1 zenith-to-horizon luminance distribution and can be generated with *gensky -c*). Following this, a Radiance octree (denoted as *sceneOC.oct*) is compiled, which contains the sky information and the full scene geometry (i.e., *scene.rad* file, previously generated in the step 3.1.4 of occupant-centered evaluations). Using the Radiance *rtrace* utility and with two ambient bounces (-ab 2, to limit computational time while including a reduced number of internal reflections), the indoor illuminance at each sensor point (from *GrdPtsH.txt*) is evaluated under overcast sky conditions, and saved as *indoorDF.txt*. Simultaneously, unobstructed horizontal illuminance is evaluated for an outdoor reference point, and saved as *outdoor_pt.txt*. The Daylight Factor is then derived for each sensor as percentage ratio of indoor to outdoor illuminance values from these two files, and saved as *daylight_factor.csv*. Data from *ann_metrics.csv* and *daylight_factor.csv* is used for grid-based visualizations.

#### 4.3.3 Workflow for occupant-centred evaluations for daylight via multichannel spectral simulations

The capacity for multi-spectral lighting simulations for daylight, i.e., raytracing across 81 channels corresponding to 5 nm spectral bins between 380 and 780 nm, leverages upon Radiance's 3-channel simulation capacities, which is then expanded into 27 times separate evaluations within the pipeline. To support this extension, the spectral emissions of daylight (as source) for each hour, as well as the reflectance and transmittance values for each material layer, are also enhanced from 3-channel RGB values to 81-channel full spectral inputs.

##### 4.3.3.1 Exporting geometry and viewing positions

The process starts by exporting the internalized geometry into separate *.obj files within a sub-folder *./Spct/*, together with the five viewing position coordinates (ViewPts*.txt), and the *suns.rad* file. These are all generated via processes similar to those detailed in steps 3.1.1., 3.1.3., and 3.1.5. of the occupant-centred evaluations.

##### 4.3.3.2 Expanding material attributes for full-spectral evaluations

The spectral attributes for each material layer, captured either via spectrophotometers, or taken from spectral databases (such as spectralDB [11]), are numerically interpolated from their original formats into uniformized 81 bins (380 to 780 nm with bin size of 5 nm). Similar to Radiance, OWL internally resamples spectra and sets spectral values to zero outside the defined range. To use the value given at e.g., 730 nm (as provided from the measurements) for all bands above until 780 nm, the input was pre-processed assuming the 730 nm value for all bands above. The uniform 81-channel definition for each material is, thereafter, split into 27 segments, with each segment covering three subsequent channels within the overall range. This 27 times 3-channel data is, thus, used to create 27 *materials.rad* files, which are saved in each of the 27 subfolders nested under *./Spct/*, with each subfolder denoted as *./div#/* (with # ranging from 0 to 26, each corresponding to channels 1–3, 4–6, 7–9, and so forth till 78–81). Although the structure of each *materials.rad* file nested under each *./div#/* subfolder remains similar to the one created and detailed in step 3.1.2. of the occupant-centred evaluations, the three channel spectral data for each material corresponds to the data bound within a specific bin, as a subset of the 81-channel interpolations for every material. In parallel, a sky file is generated and saved within each *./div#/* subfolder.

##### 4.3.3.3 Creating daylight coefficient matrices

To correspond to the full-spectral subdivision of the 81-channel material attributes across 27 subfolders, the *.obj files corresponding to each geometric group, the ViewPts*.txt files, and the *suns.rad* file from within the *./Spct/* folder are copied to each of the 27 nested subfolders. Following this step, and sequentially within each of these subfolders, the *.obj files are converted to *.rad files and meshed to *.rtm files using the segment-specific 3-channel materials data (*materials.rad*) corresponding to each layer. Together with the *.sky files, these interim files are then converted to octrees (*scene.oct*). On the other hand, the other needed octrees, i.e., the *sceneBlack.oct* (which uses a static black material) and the *sunCoefficientsDDS.oct,* are created within each subfolder akin to step 3.1.5. of the occupant-centered evaluations. Within each of the 27 subfolders, these octrees are then translated to the three categories of daylight coefficients that are required for the 2-phase DDS evaluations: total sky (./div#/illum*), direct-only sky (./div#/illum*_B), and direct solar (./div#/cdsDDS_*) daylight coefficient matrices, which are generated for each of the 5 view vectors (*), across each of the 27 spectral segments (*./div#/*).

##### 4.3.3.4 Incorporating hourly variations in daylight spectra, while extending full-spectral inputs of the daylight source

The OWL interface allows users to define bounds for binning daylight CCT-values for the simulation case, with higher number of bins corresponding to higher consideration of the dynamicity of sky-spectra. For these, the pre-calculated sky spectra recorded for each annual hour within the *.aowl file is used for the evaluations. Based on the user-defined CCT-bins (with default bounds set at 3000, 4000, 5000, 6000, 7000, 8000 K), and also taking inputs from the CCT column of the hourly spectral sky data within the *.aowl file, a key indexing file is generated within the *./Spct/* folder, denoted as the *CCT_bin_av.csv*. The process for creating this file identifies and tabulates whether each annual hour falls into either bin: from CCT_min_ (in the *.aowl file) to bin_1_, bin_1_ to bin_2_, bin_2_ to bin_3_, and so forth from bin_max_ to the CCT_max_ value (in the *.aowl file). Following this, the average values (in Kelvin) within these bin-definitions are then considered as the average CCT value for the specific bin.

##### 4.3.3.5 Generating *.wea files corresponding to each user-defined sky-CCT bin, across each of the 27 wavelength segments across the full spectral range

Using the Radiance *epw2wea* utility, a *.wea file (i.e., *location*.wea) is generated from the specified EnergyPlus weather file, which is then split into its header (saved as *location_head.wea*) and body contents (saved as *location_mod.wea*), respectively. The *.wea file's body contents are then split into multiple instances – each a subset of the original – and with each including data for hours when the sky CCT (recorded in the *.aowl file) matches the bounds of the specific bin. Together with the common header data, each instance (for a subset of hours within the total annual evaluated hours) is then saved as *location_$.wea*, with $ corresponding to the mid-CCT value within each user-defined CCT bin, and each of these are then cloned within each of the 27 subfolders (i.e., as .*/div#/location_$.wea*).

##### 4.3.3.6 Generating sky matrices for each nested instance using colour sky definitions

Using the Radiance *gendaymtx* utility with option *-m 1* for a Tregenza subdivision, and with coloured sky (-c) corresponding to the three-channel contribution for each spectral segment (i.e., 0–26) for each CCT bin average (for example, using 3500 K as the central vale for the 3000–4000 bin), the *.wea file (such as *location_3500.wea* in this case) is translated to a sky matrix (as an interim *location.smx* file) for the nested instance. For the coloured sky definition needed to generate the sky matrix within each instance – such as for generating the interim *./div#/location.smx* from *./div#/location_$.wea* – the three-channel contribution of the daylight source are defined based on each instance. These three-channel contributions are calculated for those specific hours that correspond to those listed in the nested *.wea file, and are taken from the 81-channel daylight spectral function reconstructed using the CIE015 s0, s1 and s2 functions [22] for a specific daylight CCT, which corresponds to the bin average (e.g., 3500 K) for the specific instance (e.g., 1st, 2nd, and 3rd value of the generated spectral function for the 380–385, 385–390, 390–395 nm bins within *./div1/* subfolder, or the 4th, 5th, and 6th values of the spectral function for the 395–400, 400–405, 405–410 nm bins within the *./div2/* subfolder, and so on). The *location_B.smx* and the *location_sunM6.smx* files are also created for these nested (*./div#/CCT_$*) instances as interim files.

##### 4.3.3.7 Evaluating segments of spectral irradiance within each bin for specific hours corresponding to a sky CCT within defined CCT bounds

For each instance nested under 27 wavelength segments, and further under each user-defined CCT-averaged bins, the Radiance *dctimestep* utility is used to combine the illum*.mtx daylight coefficient with the interim *location.smx* files to generate interim annualR_*.mtx files for each viewing positions. Similarly, the interim *location_B.smx* together with the daylight coefficient matrix illum*_B.mtx is translated to an interim annualR_*B.mtx (i.e., with all black material for the direct-only component). A combination of the Radiance *dctimestep* and *rmtxop* utilities converts the interim *location_sunM6.smx* together with the daylight coefficient matrix cdsDDS_*.mtx into the interim annualR_sun_*.ill files. Subsequently, the Radiance *rmtxop* utility is again used to translate the annualR_*.mtx and annualR_*B.mtx files into interim annualR_*.ill and annualR_*B.ill files, respectively, for the nested instance. Finally, *rmtxop* is again used to execute the two-phase DDS logic, and the final result is evaluated for each orientation for the nested instance by taking the interim total hourly sky illuminance (annualR_*.ill), subtracting the coarse direct-component (annualR_*B.ill), and further adding the high-resolution direct solar illuminance (annualR_sun_*.ill) contributions to generate the interim annualR_tot*.ill files. After removing headers, the interim outcome of this nested instance is finally saved as arad_*#_$.csv (with *, #, and $ indicating the orientation, the wavelength segment, and the mid-CCT value for the evaluated hours). For example, for an instance for a North-facing orientation, within the 16th wavelength bin, for the mid-CCT value of 3500 K, the final outcome is saved for those hours under *./div16/arad_N16_3500.csv*).

##### 4.3.3.8 Rearranging annual hours, merging piece-wise irradiances, and evaluating spectral outcomes

Using the indexing key (i.e., the *CCT_bin_av.csv*), the constituents of each of the mid-CCT ($) *.csv files (i.e., arad_*#_$.csv) are merged into three-channel combinations for the entire annual hours for each of the five orientations, under each of the 27 subfolders. These are saved as ./div#/AnnRad_*#.csv files, with # denoting which of the 27 spectral segments it belongs to, and * denoting the orientation of the viewing position. Furthermore, the constituents of these *.csv files within the 27 subfolders, which now contain piece-wise irradiance values for the entire annual hours, are arranged into large arrays under the parent subdirectory (*./Spct/*), and saved as mrgSpect_n*.csv files. These files are generated separately for each viewing orientation, and each include the 81-channel spectral irradiance values (in separate columns) across each hour of the year in rows, at each observer location. The data in these files are arranged such that the first 81 columns correspond to spectral irradiance at occupied position 1 across each annual hour for the given orientation, the next 81 for position 2, and so forth. With photopic and melanopic multiplication functions, these files are thereafter converted to photopic (as Pht_*.csv) and melanopic (as Mel_*.csv) illuminance values at each point for each orientation recorded for each annual hour. For a user-defined occupied position, these annual (i.e., 1 × 8760) arrays are transposed into 365 × 24 arrays, and saved as image files that present the annual hourly melanopic illuminance for that occupied position (medi_*_graph.png) across each orientation.

#### 4.3.4 Evaluations of electric lighting complementing daylight based on requirements and optimization of the dimming controls

For the evaluation of electric lighting, a cluster-based simulation approach is used to isolate the contribution of different luminaire groups. As inputs, the three-coordinate position for each luminaire within each cluster (identified as £) is defined as a *point3d* input, while also specifying the file-path of each luminaire’s IES file.

##### 4.3.4.1 Generating *.rad files for the luminaires based on their position and type

In these evaluations, within the first step, the Radiance *ies2rad* utility is used to convert the IES photometric data into Radiance-compatible material and data files, which are then saved as *all_lum£.rad*, with £ denoting the cluster that includes all luminaires of this category.

##### 4.3.4.2 Generating octrees

These luminaires are arrayed into their specific placements using the Radiance tool *xform*, and compiled into cluster-specific octrees that are then saved as *scenelum£.oct*.

##### 4.3.4.3 Conducting three-channel simulations for electric lighting

Static point-in-time simulations are performed using Radiance’s *rtrace* by defining an ambient bounce setting of -ab 2, to include also the interreflections of the artificial light. These are saved as *resultsEL_cluster£.ill*.

##### 4.3.4.4 Translation to cluster-wise photopic illuminance over the grid-points

The resulting spectral radiance is converted into illuminance values using the standard efficacy factor of 179 and photopic weights of 0.265 (R), 0.670 (G), and 0.065(B) to yield individual *.csv datasets for each luminaire group. Effectively, *resultsEL_cluster£ _RGB.csv* stores the three-channel irradiance values, while the *resultsEL_cluster£ LUX.csv* stores the illuminance values calculated with these weights.

### 4.4 How to start

Scripts have been prepared to enable the data to be used directly for simulations in Radiance and OWL.

As the EPW weather data cannot be included for licensing reasons, the first step in the preparation process is to download the two EPW climate data weatherfiles for Innsbruck and Stuttgart, and save them in the appropriate weatherdata folders (Radiance: *office/weatherdata/* and *hall/weatherdata/*; OWL: *Supplementary_Data/weather/*). The URLs for the download pages are provided in the respective directories.

#### 4.4.1 Radiance

The shell scripts *office/_office_step00_all.sh* and hall/*hall_step00_all.sh* can be called directly to run the overall Radiance simulation for the office and hall models, respectively. These scripts subsequently call all scripts office/_office_step*.sh for the single calculation steps 01 to 20 and hall/_hall_step*.sh for the single calculation steps 01 to 17.

#### 4.4.2 OWL

The scripts *DiB_OWL_Office.gh* and *DiB_OWL_Hall.gh* can be directly opened in Grasshopper to run the overall simulations in OWL for the office and for the hall model, respectively. In addition, the *_Cached.gh versions of these files can be used to readily demonstrate the pre-simulated results for both reference cases. To do this, the provided outputs must first be unzipped and saved to *‘C:\OWL\annuowl\DiB_Ofc’* or *‘C:\OWL\annuowl\DiB_Hall’* as a prior step.

## Limitations

The two reference models comprise only two areas for daylight and lighting design, namely an office and a factory hall. If, for example, software applications were developed for specific use cases (schools, healthcare, etc.), these models would be only partially transferable and meaningful for testing purposes for such tools.

Although the BSDFs for the window systems are resolved using the spectral distribution of the window panes, the spatial distribution is not spectrally resolved itself. The generation and application of spectrally resolved BSDF data is still subject of ongoing research. Yet, its necessity in the present field of application of daylight simulation in architecture is of very minor importance, as the spectral distribution of light across scattered directions hardly changes for typical materials used in construction and, in particular, in daylighting and shading systems. Should materials be used in the future to which this does not apply, such as chameleon paints as used in the automotive sector, this will need to be reassessed, as direct sunlight would then be spectrally transmitted differently depending on the sun position.

The models serve as a fully defined basis for carrying out spectral simulations, but do not provide real measurement data from the buildings (illuminance levels, HDR images, external weather data, melanopic illuminance, spectra, etc.). In order to verify and benchmark the accuracy of spectral simulations, further – maybe also simpler – models should subsequently be provided, for which real measurement data is also available.

## Ethics Statement

All authors have read and follow the ethical requirements for publication in Data in Brief and confirm that the data collection for the current work did not involve human subjects, animal experiments, or any data collected from social media platforms.

## CRediT Author Statement

**David Geisler-Moroder:** Conceptualization, Methodology, Software, Investigation, Resources, Data curation, Formal analysis, Writing – original draft, Visualization, Project administration, Funding acquisition; **Marshal Maskarenj:** Methodology, Software, Investigation, Resources, Data curation, Formal analysis, Writing – original draft, Visualization; **Greg Ward:** Methodology, Software, Investigation, Resources, Data curation, Formal analysis, Writing – Review & Editing, Visualization; **Taoning Wang:** Resources, Data curation, Writing – Review & Editing; **Eleanor S. Lee:** Conceptualization, Writing – Review & Editing, Supervision; **Sergio Altomonte:** Writing – Review & Editing, Supervision, Funding acquisition.

## Declaration of generative AI and AI-assisted technologies in the manuscript preparation process

During the preparation of this work the authors used DeepL for translations and linguistic formulations. After using this tool, the authors reviewed and edited the content as needed and take full responsibility for the content of the published article.

## Data Availability

ZenodoReference Models for Spectral Lighting and Daylight Simulations (Original data)

## Appendix Supplementary materials

Image, application 1
