# Supplementary figures and images for: Dataset describing two reference models for full-spectral lighting and daylight simulations together with implementations for two software systems
Source: Data Brief. 2026 Jun 1;67:112922. doi: 10.1016/j.dib.2026.112922 (PMC13264115; doi:10.1016/j.dib.2026.112922)

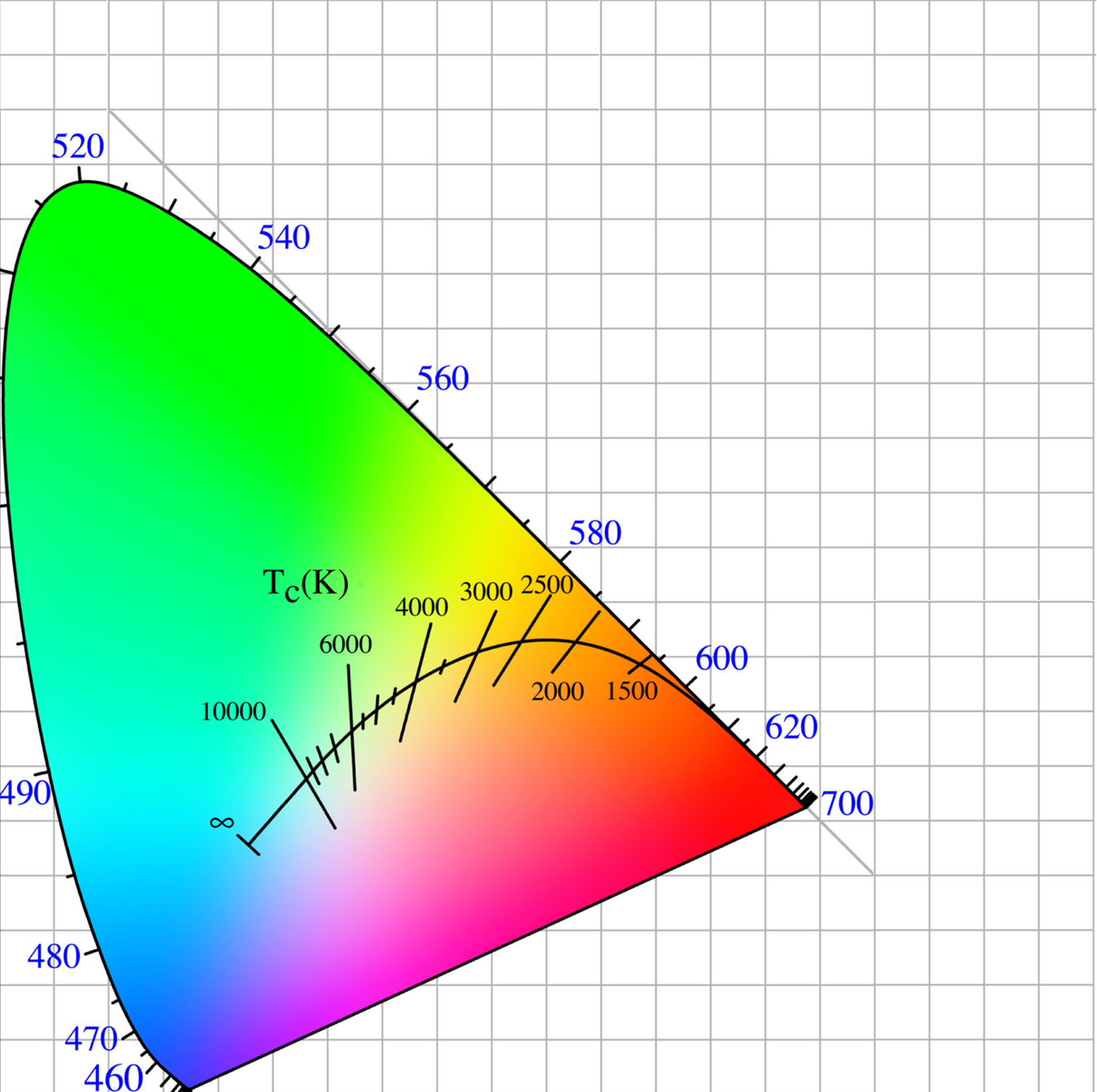

Supplement: Supplementary file 1 [file mmc1.zip › aux-data/CIE1931.png]

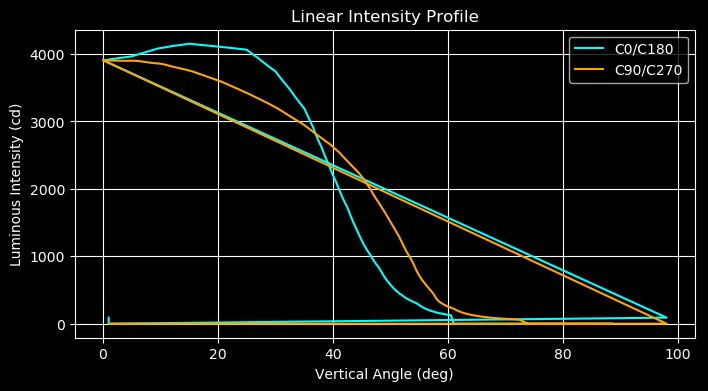

Supplement: Supplementary file 1 [file mmc1.zip › eleclight/linear_plot.png]

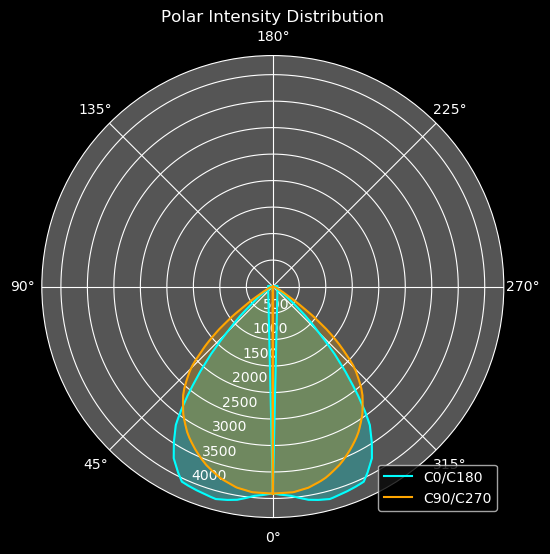

Supplement: Supplementary file 1 [file mmc1.zip › eleclight/polar_plot.png]
